# Supplementary material for: Discovery and application of insertion-deletion (INDEL) polymorphisms for QTL mapping of early life-history traits in Atlantic salmon
Source: BMC Genomics. 2010 Mar 8;11:156. doi: 10.1186/1471-2164-11-156 (PMC2838853; doi:10.1186/1471-2164-11-156)
Supplement: Additional file 2 — Information on developed 76 locus single-run INDEL panel in Atlantic salmon. Information on fluorescence labeling, primer concentrations, PCR pooling and links to alignments, INDEL motifs and GENESCAN (Burge and Karlin 1997) predictions of genes/exons are available in html format. [file 1471-2164-11-156-S2.ZIP › Additionalfile2/table3270.html]

Contig Alignment

```
Base	A B C D E F G H I J K L M N O P Q R S T U V W X Y Z a b c d e f g h i j k l m n o p q r s t u v w   
1	. . G . . . . . . . . . . . . . . . . . . . . . . . . . . . . . . . . . . . . . . . . . . . . . .    
2	. . G . . . . . . . . . . . . . . . . . . . . . . . . . . . . . . . . . . . . . . . . . . . . . .    
3	. . T . . . . . . . . . . . . . . . . . . . . . . . . . . . . . . . . . . . . . . . . . . . . . .    
4	. . G . . . . . . . . . . . . . . . . . . . . . . . . . . . . . . . . . . . . . . . . . . . . . .    
5	. . G . . . . . . . . . . . . . . . . . . . . . . . . . . . . . . . . . . . . . . . . . . . . . .    
6	. . A . . . . . . . . . . . . . . . . . . . . . . . . . . . . . . . . . . . . . . . . . . . . . .    
7	. . G . . . . . . . . . . . . . . . . . . . . . . . . . . . . . . . . . . . . . . . . . . . . . .    
8	. . T . . . . . . . . . . . . . . . . . . . . . . . . . . . . . . . . . . . . . . . . . . . . . .    
9	. . A . . . . . . . . . . . . . . . . . . . . . . . . . . . . . . . . . . . . . . . . . . . . . .    
10	. . A . . . . . . . . . . . . . . . . . . . . . . . . . . . . . . . . . . . . . . . . . . . . . .    
11	. . C . . . . . . . . . . . . . . . . . . . . . . . . . . . . . . . . . . . . . . . . . . . . . .    
12	. . T . . . . . . . . . . . . . . . . . . . . . . . . . . . . . . . . . . . . . . . . . . . . . .    
13	. . G . . . . . . . . . . . . . . . . . . . . . . . . . . . . . . . . . . . . . . . . . . . . . .    
14	. . A . . . . . . . . . . . . . . . . . . . . . . . . . . . . . . . . . . . . . . . . . . . . . .    
15	. . T . . . . . . . . . . . . . . . . . . . . . . . . . . . . . . . . . . . . . . . . . . . . . .    
16	. . A . . . . . . . . . . . . . . . . . . . . . . . . . . . . . . . . . . . . . . . . . . . . . .    
17	. . T . . . . . . . . . . . . . . . . . . . . . . . . . . . . . . . . . . . . . . . . . . . . . .    
18	. . G . . . . . . . . . . . . . . . . . . . . . . . . . . . . . . . . . . . . . . . . . . . . . .    
19	. . C . . . . . . . . . . . . . . . . . . . . . . . . . . . . . . . . . . . . . . . . . . . . . .    
20	. . G . . . . . . . . . . . . . . . . . . . . . . . . . . . . . . . . . . . . . . . . . . . . . .    
21	. . T . . . . . . . . . . . . . . . . . . . . . . . . . . . . . . . . . . . . . . . . . . . . . .    
22	. . C . . . . . . . . . . . . . . . . . . . . . . . . . . . . . . . . . . . . . . . . . . . . . .    
23	. . A . . . . . . . . . . . . . . . . . . . . . . . . . . . . . . . . . . . . . . . . . . . . . .    
24	. . A . . . . . . . . . . . . . . . . . . . . . . . . . . . . . . . . . . . . . . . . . . . . . .    
25	. . C . . . . . . . . . . . . . . . . . . . . . . . . . . . . . . . . . . . . . . . . . . . . . .    
26	. . A . . . . . . . . . . . . . . . . . . . . . . . . . . . . . . . . . . . . . . . . . . . . . .    
27	. . A . . . . . . . . . . . . . . . . . . . . . . . . . . . . . . . . . . . . . . . . . . . . . .    
28	. . T . . . . . . . . . . . . . . . . . . . . . . . . . . . . . . . . . . . . . . . . . . . . . .    
29	. . T . . . . . . . . . . . . . . . . . . . . . . . . . . . . . . . . . . . . . . . . . . . . . .    
30	. . A . . . . . . . . . . . . . . . . . . . . . . . . . . . . . . . . . . . . . . . . . . . . . .    
31	. . A . . . . . . . . . . . . . . . . . . . . . . . . . . . . . . . . . . . . . . . . . . . . . .    
32	. . G . . . . . . . . . . . . . . . . . . . . . . . . . . . . . . . . . . . . . . . . . . . . . .    
33	. . T . . . . . . . . . . . . . . . . . . . . . . . . . . . . . . . . . . . . . . . . . . . . . .    
34	. . C . . . . . . . . . . . . . . . . . . . . . . . . . . . . . . . . . . . . . . . . . . . . . .    
35	. . C . . . . . . . . . . . . . . . . . . . . . . . . . . . . . . . . . . . . . . . . . . . . . .    
36	. . T . . . . . . . . . . . . . . . . . . . . . . . . . . . . . . . . . . . . . . . . . . . . . .    
37	. . A . A . . . . . . . . . . . . . . . . . . . . . . . . . . . . . . . . . . . . . . . . . . . .    
38	. . A . A . . . . . . . . . . . . . . . . . . . . . . . . . . . . . . . . . . . . . . . . . . . .    
39	. . G . G . . . . . . . . . . . . . . . . . . . . . . . . . . . . . . . . . . . . . . . . . . . .    
40	. . T . T . . . . . . . . . . . . . . . . . . . . . . . . . . . . . . . . . . . . . . . . . . . .    
41	. . G . G . . . . . . . . . . . . . . . . . . . . . . . . . . . . . . . . . . . . . . . . . . . .    
42	. . A . A . . . . . . . . . . . . . . . . . . . . . . . . . . . . . . . . . . . . . . . . . . . .    
43	. . A . A . . . . . . . . . . . . . . . . . . . . . . . . . . . . . . . . . . . . . . . . . . . .    
44	. . A . A . . . . . . . . . . . . . . . . . . . . . . . . . . . . . . . . . . . . . . . . . . . .    
45	. . C . C . . . . . . . . . . . . . . . . . . . . . . . . . . . . . . . . . . . . . . . . . . . .    
46	. . G . G . . . . . . . . . . . . . . . . . . . . . . . . . . . . . . . . . . . . . . . . . . . .    
47	. . C . C . . . . . . . . . . . . . . . . . . . . . . . . . . . . . . . . . . . . . . . . . . . .    
48	. . C . C . . . . . . . . . . . . . . . . . . . . . . . . . . . . . . . . . . . . . . . . . . . .    
49	. . T . T . . . . . . . . . . . . . . . . . . . . . . . . . . . . . . . . . . . . . . . . . . . .    
50	. . T . T . . . . . . . . . . . . . . . . . . . . . . . . . . . . . . . . . . . . . . . . . . . .    
51	. . G . G . . . . . . . . . . . . . . . . . . . . . . . . . . . . . . . . . . . . . . . . . . . .    
52	. . C . C . . . . . . . . . . . . . . . . . . . . . . . . . . . . . . . . . . . . . . . . . . . .    
53	. . G . G . . . . . . . . . . . . . . . . . . . . . . . . . . . . . . . . . . . . . . . . . . . .    
54	. . T . T . . . . . . . . . . . . . . . . . . . . . . . . . . . . . . . . . . . . . . . . . . . .    
55	. . T . T . . . . . . . . . . . . . . . . . . . . . . . . . . . . . . . . . . . . . . . . . . . .    
56	. . T . T . . . . . . . . . . . . . . . . . . . . . . . . . . . . . . . . . . . . . . . . . . . .    
57	. . C . C . . . . . . . . . . . . . . . . . . . . . . . . . . . . . . . . . . . . . . . . . . . .    
58	. . A . A . . . . . . . . . . . . . . . . . . . . . . . . . . . . . . . . . . . . . . . . . . . .    
59	. . T . T . . . . . . . . . . . . . . . . . . . . . . . . . . . . . . . . . . . . . . . . . . . .    
60	. . A . A . . . . . . . . . . . . . . . . . . . . . . . . . . . . . . . . . . . . . . . . . . . .    
61	. . G . G . . . . . . . . . . . . . . . . . . . . . . . . . . . . . . . . . . . . . . . . . . . .    
62	. . C . C . . . . . . . . . . . . . . . . . . . . . . . . . . . . . . . . . . . . . . . . . . . .    
63	. . A . A . . . . . . . . . . . . . . . . . . . . . . . . . . . . . . . . . . . . . . . . . . . .    
64	. . A . A . . . . . . . . . . . . . . . . . . . . . . . . . . . . . . . . . . . . . . . . . . . .    
65	. . C . C . . . . . . . . . . . . . . . . . . . . . . . . . . . . . . . . . . . . . . . . . . . .    
66	. . T . T . . . . . . . . . . . . . . . . . . . . . . . . . . . . . . . . . . . . . . . . . . . .    
67	. . C . C . . . . . . . . . . . . . . . . . . . . . . . . . . . . . . . . . . . . . . . . . . . .    
68	. . A . A . . . . . . . . . . . . . . . . . . . . . . . . . . . . . . . . . . . . . . . . . . . .    
69	. . A . A . . . . . . . . . . . . . . . . . . . . . . . . . . . . . . . . . . . . . . . . . . . .    
70	. . A . A . . . . . . . . . . . . . . . . . . . . . . . . . . . . . . . . . . . . . . . . . . . .    
71	. . A . A . . . . . . . . . . . . . . . . . . . . . . . . . . . . . . . . . . . . . . . . . . . .    
72	. . A . A . . . . . . . . . . . . . . . . . . . . . . . . . . . . . . . . . . . . . . . . . . . .    
73	. . T . T . . . . . . . . . . . . . . . . . . . . . . . . . . . . . . . . . . . . . . . . . . . .    
74	. . A . A . . . . . . . . . . . . . . . . . . . . . . . . . . . . . . . . . . . . . . . . . . . .    
75	. . G . G . . . . . . . . . . . . . . . . . . . . . . . . . . . . . . . . . . . . . . . . . . . .    
76	. . C . C . . . . . . . . . . . . . . . . . . . . . . . . . . . . . . . . . . . . . . . . . . . .    
77	. . A . A . . . . . . . . . . . . . . . . . . . . . . . . . . . . . . . . . . . . . . . . . . . .    
78	. . G . G . . . . . . . . . . . . . . . . . . . . . . . . . . . . . . . . . . . . . . . . . . . .    
79	. . A . A . . . . . . . . . . . . . . . . . . . . . . . . . . . . . . . . . . . . . . . . . . . .    
80	. . A . A . . . . . . . . . . . . . . . . . . . . . . . . . . . . . . . . . . . . . . . . . . . .    
81	. . G . G . . . . . . . . . . . . . . . . . . . . . . . . . . . . . . . . . . . . . . . . . . . .    
82	. . G . G . . . . . . . . . . . . . . . . . . . . . . . . . . . . . . . . . . . . . . . . . . . .    
83	. . G . G . . . . . . . . . . . . . . . . . . . . . . . . . . . . . . . . . . . . . . . . . . . .    
84	. . G . G . . . . . . . . . . . . . . . . . . . . . . . . . . . . . . . . . . . . . . . . . . . .    
85	. . A . A . . . . . . . . . . . . . . . . . . . . . . . . . . . . . . . . . . . . . . . . . . . .    
86	. . G . G . . . . . . . . . . . . . . . . . . . . . . . . . . . . . . . . . . . . . . . . . . . .    
87	. . A . A . . . . . . . . . . . . . . . . . . . . . . . . . . . . . . . . . . . . . . . . . . . .    
88	. . C . C . . . . . . . . . . . . . . . . . . . . . . . . . . . . . . . . . . . . . . . . . . . .    
89	. . A . A . . . . . . . . . . . . . . . . . . . . . . . . . . . . . . . . . . . . . . . . . . . .    
90	. . G . G . . . . . . . . . . . . . . . . . . . . . . . . . . . . . . . . . . . . . . . . . . . .    
91	. . C . C . . . . . . . . . . . . . . . . . . . . . . . . . . . . . . . . . . . . . . . . . . . .    
92	. . G . G . . . . . . . . . . . . . . . . . . . . . . . . . . . . . . . . . . . . . . . . . . . .    
93	. . A . A . . . . . . . . . . . . . . . . . . . . . . . . . . . . . . . . . . . . . . . . . . . .    
94	. . A . A . . . . . . . . . . . . . . . . . . . . . . . . . . . . . . . . . . . . . . . . . . . .    
95	. . C . C . . . . . . . . . . . . . . . . . . . . . . . . . . . . . . . . . . . . . . . . . . . .    
96	. . A . A . . . . . . . . . . . . . . . . . . . . . . . . . . . . . . . . . . . . . . . . . . . .    
97	. . A . A . . . . . . . . . . . . . . . . . . . . . . . . . . . . . . . . . . . . . . . . . . . .    
98	. . G . G . . . . . . . . . . . . . . . . . . . . . . . . . . . . . . . . . . . . . . . . . . . .    
99	. . G . G . . . . . . . . . . . . . . . . . . . . . . . . . . . . . . . . . . . . . . . . . . . .    
100	. . G . G . . . . . . . . . . . . . . . . . . . . . . . . . . . . . . . . . . . . . . . . . . . .    
101	. . C . C . . . . . . . . . . . . . . . . . . . . . . . . . . . . . . . . . . . . . . . . . . . .    
102	. . A . A . . . . . . . . . . . . . . . . . . . . . . . . . . . . . . . . . . . . . . . . . . . .    
103	. . T . T . . . . . . . . . . . . . . . . . . . . . . . . . . . . . . . . . . . . . . . . . . . .    
104	. . T . T . . . . . . . . . . . . . . . . . . . . . . . . . . . . . . . . . . . . . . . . . . . .    
105	. . G . G . . . . . . . . . . . . . . . . . . . . . . . . . . . . . . . . . . . . . . . . . . . .    
106	. . T . T . . . . . . . . . . . . . . . . . . . . . . . . . . . . . . . . . . . . . . . . . . . .    
107	. . T . T . . . . . . . . . . . . . . . . . . . . . . . . . . . . . . . . . . . . . . . . . . . .    
108	. . C . C . . . . . . . . . . . . . . . . . . . . . . . . . . . . . . . . . . . . . . . . . . . .    
109	. . A . A . . . . . . . . . . . . . . . . . . . . . . . . . . . . . . . . . . . . . . . . . . . .    
110	. . T . T . . . . . . . . . . . . . . . . . . . . . . . . . . . . . . . . . . . . . . . . . . . .    
111	. . A . A . . . . . . . . . . . . . . . . . . . . . . . . . . . . . . . . . . . . . . . . . . . .    
112	. . T . T . . . . . . . . . . . . . . . . . . . . . . . . . . . . . . . . . . . . . . . . . . . .    
113	. . T . T . . . . . . . . . . . . . . . . . . . . . . . . . . . . . . . . . . . . . . . . . . . .    
114	. . G . G . . . . . . . . . . . . . . . . . . . . . . . . . . . . . . . . . . . . . . . . . . . .    
115	. . C . C . . . . . . . . . . . . . . . . . . . . . . . . . . . . . . . . . . . . . . . . . . . .    
116	. . T . T . . . . . . . . . . . . . . . . . . . . . . . . . . . . . . . . . . . . . . . . . . . .    
117	. . G . G . . . . . . . . . . . . . . . . . . . . . . . . . . . . . . . . . . . . . . . . . . . .    
118	. . G . G . . . . . . . . . . . . . . . . . . . . . . . . . . . . . . . . . . . . . . . . . . . .    
119	. . A . A . . . . . . . . . . . . . . . . . . . . . . . . . . . . . . . . . . . . . . . . . . . .    
120	. . G . G . . . . . . . . . . . . . . . . . . . . . . . . . . . . . . . . . . . . . . . . . . . .    
121	. . A . A . . . . . . . . . . . . . . . . . . . . . . . . . . . . . . . . . . . . . . . . . . . .    
122	. . T . T . . . . . . . . . . . . . . . . . . . . . . . . . . . . . . . . . . . . . . . . . . . .    
123	. . A . A . . . . . . . . . . . . . . . . . . . . . . . . . . . . . . . . . . . . . . . . . . . .    
124	. . G . G . . . . . . . . . . . . . . . . . . . . . . . . . . . . . . . . . . . . . . . . . . . .    
125	. . A . A . . . . . . . . . . . . . . . . . . . . . . . . . . . . . . . . . . . . . . . . . . . .    
126	. . C . C . . . . . . . . . . . . . . . . . . . . . . . . . . . . . . . . . . . . . . . . . . . .    
127	. . A . A . . . . . . . . . . . . . . . . . . . . . . . . . . . . . . . . . . . . . . . . . . . .    
128	. . G . G . . . . . . . . . . . . . . . . . . . . . . . . . . . . . . . . . . . . . . . . . . . .    
129	. . T . T . . . . . . . . . . . . . . . . . . . . . . . . . . . . . . . . . . . . . . . . . . . .    
130	. . G . G . . . . . . . . . . . . . . . . . . . . . . . . . . . . . . . . . . . . . . . . . . . .    
131	. . G . G . . . . . . . . . . . . . . . . . . . . . . . . . . . . . . . . . . . . . . . . . . . .    
132	. . G . G . . . . . . . . . . . . . . . . . . . . . . . . . . . . . . . . . . . . . . . . . . . .    
133	. . G . G . . . . . . . . . . . . . . . . . . . . . . . . . . . . . . . . . . . . . . . . . . . .    
134	. . C . C . . . . . . . . . . . . . . . . . . . . . . . . . . . . . . . . . . . . . . . . . . . .    
135	. . T . T . . . . . . . . . . . . . . . . . . . . . . . . . . . . . . . . . . . . . . . . . . . .    
136	. . G . G . . . . . . . . . . . . . . . . . . . . . . . . . . . . . . . . . . . . . . . . . . . .    
137	. . G . G . . . . . . . . . . . . . . . . . . . . . . . . . . . . . . . . . . . . . . . . . . . .    
138	. . A . A . . . . . . . . . . . . . . . . . . . . . . . . . . . . . . . . . . . . . . . . . . . .    
139	. . C . C . . . . . . . . . . . . . . . . . . . . . . . . . . . . . . . . . . . . . . . . . . . .    
140	. . G . G . . . . . . . . . . . . . . . . . . . . . . . . . . . . . . . . . . . . . . . . . . . .    
141	. . G . G . . . . . . . . . . . . . . . . . . . . . . . . . . . . . . . . . . . . . . . . . . . .    
142	. . C . C . . . . . . . . . . . . . . . . . . . . . . . . . . . . . . . . . . . . . . . . . . . .    
143	. . A . A . . . . . . . . . . . . . . . . . . . . . . . . . . . . . . . . . . . . . . . . . . . .    
144	. . T . T . . . . . . . . . . . . . . . . . . . . . . . . . . . . . . . . . . . . . . . . . . . .    
145	. . A . A . . . . . . . . . . . . . . . . . . . . . . . . . . . . . . . . . . . . . . . . . . . .    
146	. . C . C . . . . . . . . . . . . . . . . . . . . . . . . . . . . . . . . . . . . . . . . . . . .    
147	. . T . T . . . . . . . . . . . . . . . . . . . . . . . . . . . . . . . . . . . . . . . . . . . .    
148	. . T . T . . . . . . . . . . . . . . . . . . . . . . . . . . . . . . . . . . . . . . . . . . . .    
149	. . T . T . . . . . . . . . . . . . . . . . . . . . . . . . . . . . . . . . . . . . . . . . . . .    
150	. . A . A . . . . . . . . . . . . . . . . . . . . . . . . . . . . . . . . . . . . . . . . . . . .    
151	. . T . T . . . . . . . . . . . . . . . . . . . . . . . . . . . . . . . . . . . . . . . . . . . .    
152	. . T . T . . . . . . . . . . . . . . . . . . . . . . . . . . . . . . . . . . . . . . . . . . . .    
153	. . A . A . . . . . . . . . . . . . . . . . . . . . . . . . . . . . . . . . . . . . . . . . . . .    
154	. . C . C . . . . . . . . . . . . . . . . . . . . . . . . . . . . . . . . . . . . . . . . . . . .    
155	. . T . T . . . . . . . . . . . . . . . . . . . . . . . . . . . . . . . . . . . . . . . . . . . .    
156	. . A . A . . . . . . . . . . . . . . . . . . . . . . . . . . . . . . . . . . . . . . . . . . . .    
157	. . G . G . . . . . . . . . . . . . . . . . . . . . . . . . . . . . . . . . . . . . . . . . . . .    
158	. . T . T . . . . . . . . . . . . . . . . . . . . . . . . . . . . . . . . . . . . . . . . . . . .    
159	. . A . A . . . . . . . . . . . . . . . . . . . . . . . . . . . . . . . . . . . . . . . . . . . .    
160	. . A . A . . . . . . . . . . . . . . . . . . . . . . . . . . . . . . . . . . . . . . . . . . . .    
161	. . C . C . . . . . . . . . . . . . . . . . . . . . . . . . . . . . . . . . . . . . . . . . . . .    
162	. . A . A . . . . . . . . . . . . . . . . . . . . . . . . . . . . . . . . . . . . . . . . . . . .    
163	. . T . T . . . . . . . . . . . . . . . . . . . . . . . . . . . . . . . . . . . . . . . . . . . .    
164	. . T . T . . . . . . . . . . . . . . . . . . . . . . . . . . . . . . . . . . . . . . . . . . . .    
165	. . G . G . . . . . . . . . . . . . . . . . . . . . . . . . . . . . . . . . . . . . . . . . . . .    
166	. . A . A . . . . . . . . . . . . . . . . . . . . . . . . . . . . . . . . . . . . . . . . . . . .    
167	. . G . G . G . . . . . . . . . . . . . . . . . . . . . . . . . . . . . . . . . . . . . . . . . .    
168	. . G . G . G . . . . . . . . . . . . . . . . . . . . . . . . . . . . . . . . . . . . . . . . . .    
169	. . G . G . G . . . . . . . . . . . . . . . . . . . . . . . . . . . . . . . . . . . . . . . . . .    
170	. . G . G . G . . . . . . . . . . . . . . . . . . . . . . . . . . . . . . . . . . . . . . . . . .    
171	. . T . T . T . . . . . . . . . . . . . . . . . . . . . . . . . . . . . . . . . . . . . . . . . .    
172	. . T . T . T . . . . . . . . . . . . . . . . . . . . . . . . . . . . . . . . . . . . . . . . . .    
173	. . T . T . T . . . . . . . . . . . . . . . . . . . . . . . . . . . . . . . . . . . . . . . . . .    
174	. . C . C . C . . . . . . . . . . . . . . . . . . . . . . . . . . . . . . . . . . . . . . . . . .    
175	. . T . T . T . . . . . . . . . . . . . . . . . . . . . . . . . . . . . . . . . . . . . . . . . .    
176	. . G . G . G . . . . . . . . . . . . . . . . . . . . . . . . . . . . . . . . . . . . . . . . . .    
177	. . A . A . A . . . . . . . . . . . . . . . . . . . . . . . . . . . . . . . . . . . . . . . . . .    
178	. . A . A . A . . . . . . . . . . . . . . . . . . . . . . . . . . . . . . . . . . . . . . . . . .    
179	. . G . G . G . . . . . . . . . . . . . . . . . . . . . . . . . . . . . . . . . . . . . . . . . .    
180	. . T . T . T . . . . . . . . . . . . . . . . . . . . . . . . . . . . . . . . . . . . . . . . . .    
181	. . T . T . T . . . . . . . . . . . . . . . . . . . . . . . . . . . . . . . . . . . . . . . . . .    
182	. . C . C . C . . . . . . . . . . . . . . . . . . . . . . . . . . . . . . . . . . . . . . . . . .    
183	. . C . C . C . . . . . . . . . . . . . . . . . . . . . . . . . . . . . . . . . . . . . . . . . .    
184	. . T . T . T . . . . . . . . . . . . . . . . . . . . . . . . . . . . . . . . . . . . . . . . . .    
185	. . T . T . T . . . . . . . . . . . . . . . . . . . . . . . . . . . . . . . . . . . . . . . . . .    
186	. . T . T . T . . . . . . . . . . . . . . . . . . . . . . . . . . . . . . . . . . . . . . . . . .    
187	. . T . T . T . . . . . . . . . . . . . . . . . . . . . . . . . . . . . . . . . . . . . . . . . .    
188	. . C . C . C . . . . . . . . . . . . . . . . . . . . . . . . . . . . . . . . . . . . . . . . . .    
189	. . A . A . A . . . . . . . . . . . . . . . . . . . . . . . . . . . . . . . . . . . . . . . . . .    
190	. . T . T . T . . . . . . . . . . . . . . . . . . . . . . . . . . . . . . . . . . . . . . . . . .    
191	. . G . G . G . . . . . . . . . . . . . . . . . . . . . . . . . . . . . . . . . . . . . . . . . .    
192	. . T . T . T . . . . . . . . . . . . . . . . . . . . . . . . . . . . . . . . . . . . . . . . . .    
193	. . A . A . A . . . . . . . . . . . . . . . . . . . . . . . . . . . . . . . . . . . . . . . . . .    
194	. . A . A . A . . . . . . . . . . . . . . . . . . . . . . . . . . . . . . . . . . . . . . . . . .    
195	. . G . G . G . . . . . . . . . . . . . . . . . . . . . . . . . . . . . . . . . . . . . . . . . .    
196	. . A . A . A . . . . . . . . . . . . . . . . . . . . . . . . . . . . . . . . . . . . . . . . . .    
197	. . C . C . C . . . . . . . . . . . . . . . . . . . . . . . . . . . . . . . . . . . . . . . . . .    
198	. . G . G . G . . . . . . . . . . . . . . . . . . . . . . . . . . . . . . . . . . . . . . . . . .    
199	. . C . C . C . . . . . . . . . . . . . . . . . . . . . . . . . . . . . . . . . . . . . . . . . .    
200	. . T . T . T . . . . . . . . . . . . . . . . . . . . . . . . . . . . . . . . . . . . . . . . . .    
201	. . T . T . T . . . . . . . . . . . . . . . . . . . . . . . . . . . . . . . . . . . . . . . . . .    
202	. . T . T . T . . . . . . . . . . . . . . . . . . . . . . . . . . . . . . . . . . . . . . . . . .    
203	. . T . T . T . . . . . . . . . . . . . . . . . . . . . . . . . . . . . . . . . . . . . . . . . .    
204	. . C . C . C . . . . . . . . . . . . . . . . . . . . . . . . . . . . . . . . . . . . . . . . . .    
205	. . T . T . T . . . . . . . . . . . . . . . . . . . . . . . . . . . . . . . . . . . . . . . . . .    
206	. . T . T . T . . . . . . . . . . . . . . . . . . . . . . . . . . . . . . . . . . . . . . . . . .    
207	. . T . T . T . . . . . . . . . . . . . . . . . . . . . . . . . . . . . . . . . . . . . . . . . .    
208	. . T . T . T . . . . . . . . . . . . . . . . . . . . . . . . . . . . . . . . . . . . . . . . . .    
209	. . T . T . T . . . . . . . . . . . . . . . . . . . . . . . . . . . . . . . . . . . . . . . . . .    
210	. . T . T . T . . . . . . . . . . . . . . . . . . . . . . . . . . . . . . . . . . . . . . . . . .    
211	. . G . G . G . . . . . . . . . . . . . . . . . . . . . . . . . . . . . . . . . . . . . . . . . .    
212	. . T . T . T . . . . . . . . . . . . . . . . . . . . . . . . . . . . . . . . . . . . . . . . . .    
213	. . C . C . C . . . . . . . . . . . . . . . . . . . . . . . . . . . . . . . . . . . . . . . . . .    
214	. . G . G . G . . . . . . . . . . . . . . . . . . . . . . . . . . . . . . . . . . . . . . . . . .    
215	. . T . T . T . . . . . . . . . . . . . . . . . . . . . . . . . . . . . . . . . . . . . . . . . .    
216	. . T . T . T . . . . . . . . . . . . . . . . . . . . . . . . . . . . . . . . . . . . . . . . . .    
217	. . T . T . T . . . . . . . . . . . . . . . . . . . . . . . . . . . . . . . . . . . . . . . . . .    
218	. . G . G . G . . . . . . . . . . . . . . . . . . . . . . . . . . . . . . . . . . . . . . . . . .    
219	. . G . G . G . . . . . . . . . . . . . . . . . . . . . . . . . . . . . . . . . . . . . . . . . .    
220	. . C . C . C . . . . . . . . . . . . . . . . . . . . . . . . . . . . . . . . . . . . . . . . . .    
221	. . T . T . T . . . . . . . . . . . . . . . . . . . . . . . . . . . . . . . . . . . . . . . . . .    
222	. . A . A . A . . A . . . . . . . . . . . . . . . . . . . . . . . . . . . . . . . . . . . . . . .    
223	. . G . G . G . . G . . . . . . . . . . . . . . . . . . . . . . . . . . . . . . . . . . . . . . .    
224	. . G . G . G . . G . . . . . . . . . . . . . . . . . . . . . . . . . . . . . . . . . . . . . . .    
225	. . C . C . C . . C . . . . . . . . . . . . . . . . . . . . . . . . . . . . . . . . . . . . . . .    
226	. . G . G . G . . G . . . . . . . . . . . . . . . . . . . . . . . . . . . . . . . . . . . . . . .    
227	. . G . G . G . . G . . . . . . . . . . . . . . . . . . . . . . . . . . . . . . . . . . . . . . .    
228	. . A . A . A . . A . . . . . . . . . . . . . . . . . . . . . . . . . . . . . . . . . . . . . . .    
229	. . A . A . A . . A . . . . . . . . . . . . . . . . . . . . . . . . . . . . . . . . . . . . . . .    
230	. . C . C . C . . C . . . . . . . . . . . . . . . . . . . . . . . . . . . . . . . . . . . . . . .    
231	. . G . G . G . . G . . . . . . . . . . . . . . . . . . . . . . . . . . . . . . . . . . . . . . .    
232	. . A . C . C . . C . . . . . . . . . . . . . . . . . . . . . . . . . . . . . . . . . . . . . . .    
233	. . A . A . A . . A . . . . . . . . . . . . . . . . . . . . . . . . . . . . . . . . . . . . . . .    
234	. . T . T . T . . T . . . . . . . . . . . . . . . . . . . . . . . . . . . . . . . . . . . . . . .    
235	. . G G G . G G . G . . . . . . . . . . . . . . . . . . . . . . . . . . . . . . . . . . . . . . .    
236	. . A A A . A A . A . . . . . . . . . . . . . . . . . . . . . . . . . . . . . . . . . . . . . . .    
237	. . C C C . C C . C . . . . . . . . . . . . . . . . . . . . . . . . . . . . . . . . . . . . . . .    
238	. . C C C . C C . C . . . . . . . . . . . . . . . . . . . . . . . . . . . . . . . . . . . . . . .    
239	. . A A A . A A . A . . . . . . . . . . . . . . . . . . . . . . . . . . . . . . . . . . . . . . .    
240	. . T T T . T T . T . . . . . . . . . . . . . . . . . . . . . . . . . . . . . . . . . . . . . . .    
241	. . T T T . T T . T . . . . . . . . . . . . . . . . . . . . . . . . . . . . . . . . . . . . . . .    
242	. . A A A . A A . A . . . . . . . . . . . . . . . . . . . . . . . . . . . . . . . . . . . . . . .    
243	. . T T T . T T . T . . . . . . . . . . . . . . . . . . . . . . . . . . . . . . . . . . . . . . .    
244	. . T T T . T T . T . . . . . . . . . . . . . . . . . . . . . . . . . . . . . . . . . . . . . . .    
245	. . T T T . T T . T . . . . . . . . . . . . . . . . . . . . . . . . . . . . . . . . . . . . . . .    
246	. . T T T . T T . T . . . . . . . . . . . . . . . . . . . . . . . . . . . . . . . . . . . . . . .    
247	. . G G G . G G . G . . . . . . . . . . . . . . . . . . . . . . . . . . . . . . . . . . . . . . .    
248	. . T T T . T T . T . . . . . . . . . . . . . . . . . . . . . . . . . . . . . . . . . . . . . . .    
249	. . G G G . G G . G . . . . . . . . . . . . . . . . . . . . . . . . . . . . . . . . . . . . . . .    
250	. . C C C . C C . C . . . . . . . . . . . . . . . . . . . . . . . . . . . . . . . . . . . . . . .    
251	. . C C C . C C . C . . . . . . . . . . . . . . . . . . . . . . . . . . . . . . . . . . . . . . .    
252	. . A A A . A A . A . . . . . . . . . . . . . . . . . . . . . . . . . . . . . . . . . . . . . . .    
253	. . C C C . C C . C . . . . . . . . . . . . . . . . . . . . . . . . . . . . . . . . . . . . . . .    
254	. . A A A . A A . A . . . . . . . . . . . . . . . . . . . . . . . . . . . . . . . . . . . . . . .    
255	. . C C C . C C . C . . . . . . . . . . . . . . . . . . . . . . . . . . . . . . . . . . . . . . .    
256	. . G G G . G G . G . . . . . . . . . . . . . . . . . . . . . . . . . . . . . . . . . . . . . . .    
257	. . G G G . G G . G . . . . . . . . . . . . . . . . . . . . . . . . . . . . . . . . . . . . . . .    
258	. . A A A . A A . A . . . . . . . . . . . . . . . . . . . . . . . . . . . . . . . . . . . . . . .    
259	. . A A A . A A . A . . . . A . . . . . . . . . . . . . . . . . . . . . . . . . . . . . . . . . .    
260	. . C C C . C C . C . . . . C . . . . . . . . . . . . . . . . . . . . . . . . . . . . . . . . . .    
261	. . T T T . T T . T . . . . T . . . . . . . . . . . . . . . . . . . . . . . . . . . . . . . . . .    
262	. . T T T . T T . T . . . . T . . . . . . . . . . . . . . . . . . . . . . . . . . . . . . . . . .    
263	. . G G G . G G . G . . . . G G . . . . . . . . . . . . . . . . . . . . . . . . . . . . . . . . .    
264	. . A A A . A A . A . . . . A A . . . . . . . . . . . . . . . . . . . . . . . . . . . . . . . . .    
265	. . A A A . A A . A . . . . A A . . . . . . . . . . . . . . . . . . . . . . . . . . . . . . . . .    
266	. . G G G . G G . G . . . . G G . . . . . . . . . . . . . . . . . . . . . . . . . . . . . . . . .    
267	. . T T T . T T . T . . . . T T . . . . . . . . . . . . . . . . . . . . . . . . . . . . . . . . .    
268	. . T T T . T T . T . . . . T T . . . . . . . . . . . . . . . . . . . . . . . . . . . . . . . . .    
269	. . G G G . G G . G . . . . G G . . . . . . . . . . . . . . . . . . . . . . . . . . . . . . . . .    
270	. . A A A . A A . A . . . . A A . . . . . . . . . . . . . . . . . . . . . . . . . . . . . . . . .    
271	. . G G G . G G . G . . . . G G . . . . . . . . . . . . . . . . . . . . . . . . . . . . . . . . .    
272	. . G G G . G G . G . . . . G G . . . . . . . . . . . . . . . . . . . . . . . . . . . . . . . . .    
273	. . C C C . C C . C . . . . C C . . . . . . . . . . . . . . . . . . . . . . . . . . . . . . . . .    
274	A . A A A . A A . A . . . . A A . . . . . . . . . . . . . . . . . . . . . . . . . . . . . . . . .    
275	A . A A A . A A . A . . . . A A . . . . . . . . . . . . . . . . . . . . . . . . . . . . . . . . .    
276	A . A A A . A A . A . . . . A A . . . . . . . . . . . . . . . . . . . . . . . . . . . . . . . . .    
277	A . A A A . A A . A . . . . A A A . . . . . . . . . . . . . . . . . . . . . . . . . . . . . . . .    
278	G . G G G . G G . G . . . . G G G . . . . . . . . . . . . . . . . . . . . . . . . . . . . . . . .    
279	A . A A A . A A . A . . . . A A A . . . . . . . . . . . . . . . . . . . . . . . . . . . . . . . .    
280	T . T T T . T T . T . . . . T T T . . . . . . . . . . . . . . . . . . . . . . . . . . . . . . . .    
281	C . C C C . C C . C . . . . C C C . . . . . . . . . . . . . . . . . . . . . . . . . . . . . . . .    
282	A A A A A A A A . A A . . . A A A . . . . . . . . . A . . . . . . . . . . . . . . . . . . . . . .    
283	A A A A A A A A . A A . . . A A A . . . . . . . . . A . . . . . . . . . . . . . . . . . . . . . .    
284	T T T T T T T T . T T . . . T T T . . . . . . . . . T . . . . . . . . . . . . . . . . . . . . . .    
285	C C C C C C C C . C C . . . C C C . . . . . . . . . C . . . . . . . . . . . . . . . . . . . . . .    
286	A A A A A A A A . A A . . . A A A . . A . . . . . . A . . . . . . . . . . . . . . . . . . . . . .    
287	A A A A A A A A . A A . . . A A A . . A . A . . . . A . . . . . . . . . . . . . . . . . . . . . .    
288	G G G G G G G G . G G . . . G G G . . G . G . . . . G . . . . . . . . . . . . . . . . . . . . . .    
289	T T T T T T T T . T T . . . T T T . . T . T . . . . T . . . . . . . . . . . . . . . . . . . . . .    
290	A A A A A A A A . A A . A . A A A . . A . A . A . . A . . . . . . A . . . . . . . . . . . . . . .    
291	A A A A A A A A . A A . A . A A A . . A . A . A . . A . . . . . . A . . . . . . . . . . . . . . .    
292	A A A A A A A A . A A . A . A A A . . A . A . A . . A . . . . . . A . . . . . . . . . . . . . . .    
293	C C C C C C C C . C C . C . C C C . . C . C . C . . C . . . . . . C . . . . . . . . . . . . . . .    
294	T T T T T T T T . T T . T . T T T . . T . T . T . . T . . . . . . T . . . . . . . . . . . . . . .    
295	A A A A A A A A . A A . A . A A A . . A . A . A . . A . . . . . . A . . . . . . . . . . . . . . .    
296	A A A A A A A A . A A . A . A A A . . A . A . A . . A . . . . . . A . . . . . . . . . . . . . . .    
297	A A A A A A A A . A A . A . A A A . . A . A . A . . A . . . . . . A . . . . A . . . . . . . . . .    
298	G G G G G G G G . G G . G . G G G . . G . G . G . . G . . . . . . G . . . . G . . . . . G . . . .    
299	T T T T T T T T . T T . T . T T T . . T . T . T . . T . . . . . . T . . . . T . . . . T T . . . .    
300	A A A A A A A A . A A . A . A A A . . A . A . A . . A . . . . . . A . . . . A . . . . A A . . . .    
301	A A A A A A A A . A A . A . A A A . . A . A . A . . A . . . . . . A . . . . A . . . . A A . . . .    
302	C C C C C C C C . C C . C . C C C . . C . C . C . . C . . . . . . C . . . . C . . . . C C . . . .    
303	A A A A A A A A . A A . A . A A A . . A . A . A . . A . . . . . . A . . . . A . . . . A A . . . .    
304	T T T T T T T T . T T . T . T T T . . T . T . T . . T . . . . . . T . . . . T . . . . T T . . . .    
305	C C C C C C C C . C C . C . C C C . . C . C . C . . C . . . . . . C . . . . C . . . . C C . . . .    
306	A A A A A A A A . A A . A . A A A . . A . A . A . . A . . . . . . A . . . . A . . . . A A . . . .    
307	G G G G G G G G . G G . G . G G G . . G . G . G . . G . . . . . . G . . . . G . . . . G G . . . .    
308	C C C C C C C C . C C . C . C C C . . C . C . C . . C . . . . . . C . . . . C . . . . C C . . . .    
309	A A A A A A A A . A A . A . A A A . . A . A . A . . A . . . . . . A . . . . A . . . . A A . . . .    
310	A A A A A A A A . A A . A . A A A . . A . A . A . . A . . . . . . A . . . . A . . . . A A . . . .    
311	C C C C C C C C . C C . C . C C C . . C . C . C . . C . . . . . . C . . . . C . . . . C C . . . .    
312	A A A A A A A A . A A . A . A A A . . A . A . A . . A . . . . . . A . . . . A . . . . A A . . . .    
313	A A A A A A A A . A A . A . A A A . . A . A . A . . A . . . . . . A . . . . A . . . . A A . . . .    
314	G G G G G G G G . G G . G . G G G . . G . G . G . . G . . . . . . G . . . . G . . . . G G . . . .    
315	T T T T T T T T . T T . T . T T T . . T . T . T . . T . . . . . . T . . . . T . . . . T T . . . .    
316	A A A A A A A A . A A . A . A A A . . A . A . A . . A . . . . . . A . . . . A . . . . A A . . . .    
317	A A A A A A A A . A A . A . A A A . . A . A . A . . A . . . . . . A . . . . A . . . . A A . . . .    
318	C C C C C C C C . C C . C . C C C . . C . C . C . . C . . . . . . C . . . . C . . . . C C . . . .    
319	G G G G G G G G . G G . G . G G G . . G . G . G . . G . . . . . . G . . . . G . . . . G G . . . .    
320	T T T T T T T T . T T . T . T T T . . T . T . T . . T . . . . . . T . . . . T . . . T T T . . . .    
321	T T T T T T T T . T T . T . T T T . . T . T . T . . T . . . . . . T . . . . T . . . T T T . . . .    
322	A A A A A A A A . A A . A . A A A . . A . A . A . . A . . . . . . A . . . . A . . . A A A . . . .    
323	C C C C C C C C . C C C C . C C C . . C . C . C C . C . . . . . . C . . . . C . . . C C C . . . .    
324	T T T T T T T T . T T T T . T T T . . T . T . T T . T . . . . . . T . . . . T . . . T T T . . . .    
325	A A A A A A A A . A A A A . A A A . . A . A . A A . A . . . . . . A . . . . A . . . A A A . . . .    
326	G G G G G G G G . G G G G . G G G . . G . G . G G . G . . . . . . G . . . . G . . . G G G . . . .    
327	T T T T T T T T . T T T T . T T T . . T . T . T T . T . . . . . . T . . . . T . . . T T T . . . .    
328	A A A A A A A A . A A A A . A A A . . A . A . A A . A . . . . . . A . . . . A . . . A A A . . . .    
329	A A A A A A A A . A A A A . A A A . . A . A . A A . A . . . . . . A . . . . A . . . A A A . . . .    
330	C C C C C C C C . C C C C . C C C . . C . C . C C . C . . . . . . C . . . . C . . . C C C . . . .    
331	A A A A A A A A . A A A A . A A A . . A . A . A A . A . . . . . A A . . . . A . . . A A A . . . .    
332	A A A A A A A A . A A A A . A A A . . A . A . A A . A . . . . . A A . . . . A . . . A A A . . . .    
333	C C C C C C C C . C C C C . C C C . . C . C . C C . C . . . . . C C . . . . C . . . C C C . . . .    
334	A A A A A A A A . A A A A . A A A . . A . A . A A . A . . . . . A A . . . . A . . . A A A . . . .    
335	G G G G G G G G . G G G G . G G G . . G . G . G G . G . . . . . G G . . . . G . . . G G G . . . .    
336	A A A A A A A A . A A A A . A A A . . A . A . A A . A . . . . . A A . . . . A . . . A A A . . . .    
337	T T T T T T T T . T T T T . T T T . . T . T . T T . T . . . . . T T . . . . T . . . T T T . . . .    
338	G G G G G G G G . G G G G . G G G . . G . G . G G . G . . . . G G G . . . . G . . . G G G . . . .    
339	T T T T T T T T . T T T T . T T T . . T . T . T T . T . . . . T T T . . . . T . . . T T T . . . .    
340	G G G G G G G G . G G G G . G G G . . G . G . G G . G . . . . G G G . . . . G . . . G G G G . . .    
341	G G G G G G G G . G G G G . G G G . . G . G . G G . G . . . . G G G . . . . G . . . G G G G . . .    
342	A A A A A A A A . A A A A . A A A . . A . A . A A . A . . . . A A A . . . . A . . . A A A A . . .    
343	T T T T T T T T . T T T T . T T T . . T . T . T T . T . . . . T T T . . . . T . . . T T T T . . .    
344	G G G G G G G G . G G G G . G G G . . G . G . G G . G . . . . G G G . . . . G . . . G G G G . . .    
345	A A A A A A A A . A A A A . A A A . . A . A . A A . A . . . . A A A . . . . A . . . A A A A . . .    
346	G G G G G G G G . G G G G . G G G . . G . G . G G . G . . . . G G G . . . . G . . . G G G G . . .    
347	A A A A A A A A . A A A A . A A A . . A . A . A A . A . . . . A A A . . . . A . . . A A A A . . .    
348	C C C C C C C C . C C C C . C C C . . C . C . C C . C . . . . C C C . . . . C . . . C C C C . . .    
349	G G G G G G G G . G G G G . G G G . . G . G . G G . G . . . . G G G . . . . G . . . G G G G . . .    
350	G G G G G G G G . G G G G . G G G . . G . G . G G . G . . . . G G G . . . . G . . . G G G G . . .    
351	T T T T T T T T . T T T T . T T T . . T . T . T T . T . . . . T T T . . . . T . . . T T T T . . .    
352	G G G G G G G G . G G G G . G G G . . G . G . G G . G . . . . G G G . . . . G . . . G G G G . . .    
353	T T T T T T T T . T T T T . T T T . . T . T . T T . T . . . . T T T . . . . T . . . T T T T . . .    
354	T T T T T T T T . T T T T . T T T . . T . T . T T . T . . . . T T T . . . . T . . . T T T T . . .    
355	A A A A A A A A . A A A A . A A A . . A . A . A A . A . . . . A A A . . . . A . . . A A A A . . .    
356	T T T T T T T T . T T T T . T T T . . T . T . T T . T . . . . T T T . . . . T . . . T T T T . . .    
357	A A A A A A A A . A A A A . A A A . . A . A . A A . A . . . . A A A . . . . A . . . A A A A . . .    
358	A A A A A A A A . A A A A . A A A . . A . A . A A . A . . . . A A A . . . . A . . . A A A A . . .    
359	A A A A A A A A . A A A A . A A A . . A . A . A A . A . . . . A A A . . . . A . . . A A A A . . .    
360	C C C C C C C C . C C C C . C C C . . C . C . C C . C . . . . C C C . . . . C . . . C C C C . . .    
361	G G G G G G G G . G G G G . G G G . . G . G . G G . G . . . . G G G . . . . G . . . G G G G . . .    
362	C C C C C C C C . C C C C . C C C . . C . C . C C . C . . . . C C C . . . . C . . . C C C C . . .    
363	G G G G G G G G . G G G G G G G G . . G . G . G G . G . . . . G G G . . . . G . . . G G G G . . .    
364	C C C C C C C C . C C C C C C C C . . C . C . C C . C . . . . C C C . . . . C . . . C C C C . . .    
365	A A A A A A A A . A A A A A A A A . . A . A . A A . A . . . . A A A . . . . A . . . A A A A . . .    
366	A A A A A A A A . A A A A A A A A . . A . A . A A . A . . . . A A A . . . . A . . . A A A A . . .    
367	A A A A A A A A . A A A A A A A A . . A . A . A A . A . . . . A A A . . . . A . . . A A A A . . .    
368	- - - - - - - - . - - - - - - - - . . - . - . - - . - . . . . - - - . . . . - . . . A - - - . . .    
369	T T T T T T T T . T T T T T T T T . . T . T . T T . T . . . . T T T . . . . T . . . T T T T . . .    
370	G G G G G G G G . G G G G G G G G . . G . G . G G . G . . . . G G G . . . . G . . . G G G G . . .    
371	T T T T T T T T . T T T T T T T T . . T . T . T T . T . . . . T T T . . . . T . . . T T T T . . .    
372	T T T T T T T T . T T T T T T T T . . T . T . T T . T . . . . T T T . . . . T . . . T T T T . . .    
373	T T T T T T T T . T T T T T T T T . . T . T . T T . T . . . . T T T . . . . T . . . T T T T . . .    
374	A A A A A A A A . A A A A A A A A . . A . A . A A . A . . . . A A A . . . . A . . . A A A A . . .    
375	A A A A A A A A . A A A A A A A A . . A . A . A A . A . . . . A A A . . . . A . . . A A A A . . .    
376	A A A A A A A A . A A A A A A A A . . A . A . A A . A . . . . A A A . . . . A . . . A A A A . . .    
377	A A A A A A A A . A A A A A A A A . . A . A . A A . A . . . . A A A . . . . A . . . A A A A . . .    
378	A A A A A A A A . A A A A A A A A . . A . A . A A . A . . . . A A A . . . . A . . . A A A A . . .    
379	C C C C C C C C . C C C C C C C C . . C . C . C C . C . . . . C C C . . . . C . . . C C C C . . .    
380	A A A A A A A A . A A A A A A A A . . A . A . A A . A . . . . A A A . . . . A . . . A A A A . . .    
381	C C C C C C C C C C C C C C C C C . . C . C . C C . C . . . . C C C . . . . C . . . C C C C . . .    
382	C C C C C C C C C C C C C C C C C . . C . C . C C . C . . . . C C C . . . . C . . . C C C C . . .    
383	T T T T T T T T T T T T T T T T T . . T . T . T T . T . . . . T T T . . . . T . . . T T T T . . .    
384	T T T T T T T T T T T T T T T T T . . T . T . T T . T . . . . T T T . . . . T . . . T T T T . . .    
385	T T T T T T T T T T T T T T T T T . . T . T . T T . T . . . . T T T . . . . T . . . T T T T . . .    
386	C C C C C C C C C C C C C C C C C . . C . C . C C . C . . . . C C C . . . . C . . . C C C C . . .    
387	A A A A A A A A A A A A A A A A A . . A . A . A A . A . . . . A A A . . . . A . . . A A A A . . .    
388	A A A A A A A A A A A A A A A A A . . A . A . A A . A . . . . A A A . . . . A . . . A A A A . . .    
389	A A A A A A A A A A A A A A A A A . . A . A . A A . A . . . . A A A . . . . A . . . A A A A . . .    
390	G G G G G G G G G G G G G G G G G . . G . G . G G . G . . . . G G G . . . . G . . . G G G G . . .    
391	T T T T T T T T T T T T T T T T T . . T . T . T T . T . . . . T T T . . . . T . . . T T T T . . .    
392	G G G G G G G G G G G G G G G G G . G G . G . G G . G . . . . G G G . . . . G . . . G G G G . . .    
393	G G G G G G G G G G G G G G G G G . G G . G . G G . G . . . . G G G . . . . G . . . G G G G . . .    
394	A A A A A A A A A A A A A A A A A . A A . A . A A . A . . . . A A A . . . . A . . . A A A A . . .    
395	T T T T T T T T T T T T T T T T T . T T . T . T T . T . . . . T T T . . . . T . . . T T T T . . .    
396	T T T T T T T T T T T T T T T T T . T T . T . T T . T . . . . T T T . . . . T . . . T T T T . . .    
397	C C C C C C C C C C C C C C C C C . C C . C . C C . C . . . . C C C . . . . C . . . C C C C . . .    
398	C C C C C C C C C C C C C C C C C . C C . C . C C . C . . . . C C C . . . . C . . . C C C C . . .    
399	T T T T T T T T T T T T T T T T T . T T . T . T T . T . . . . T T T . . . . T . . . T T T T . . .    
400	A A A A A A A A A A A A A A A A A . A A . A . A A . A . . . . A A A . . . . A . . . A A A A . . .    
401	T T T T T T T T T T T T T T T T T . T T . T . T T . T . . . . T T T . . . . T . . . T T T T . . .    
402	C C C C C C C C C C C C C C C C C . C C C C . C C . C . . . . C C C . . . . C . . . C C C C . . .    
403	T T T T T T T T T T T T T T T T T . T T T T . T T . T . . . . T T T . . . . T . . . T T T T . . .    
404	A A A A A A A A A A A A A A A A A . A A A A . A A . A . . . . A A A . . . . A . . . A A A A . . .    
405	T T T T T T T T T T T T T T T T T . T T T T . T T . T . . . . T T T . . . . T . . . T T T T . . .    
406	C C C C C C C C C C C C C C C C C . C C C C . C C . C . . . . C C C . . . . C . . . C C C C . . .    
407	T T T T T T T T T T T T T T T T T . T T T T . T T . T . . . T T T T . . . . T . . . T T T T . . .    
408	T T T T T T T T T T T T T T T T T . T T T T . T T . T . . . T T T T . . . . T . . . T T T T . . .    
409	G G G G G G G G G G G G G G G G G . G G G G . G G . G . . . G G G G . . . . G . . . G G G G . . .    
410	T T T T T T T T T T T T T T T T T . T T T T . T T . T . . . T T T T . . . . T . . . T T T T . . .    
411	A A A A A A A A A A A A A A A A A . A A A A . A A . A . . . A A A A . . . . A . . . A A A A . . .    
412	T T T T T T T T T T T T T T T T T . T T T T . T T . T . . . T T T T . . . . T . . . T T T T . . .    
413	A A A A A A A A A A A A A A A A A . A A A A . A A . A . . . A A A A . . . . A . . . A A A A . . .    
414	G G G G G G G G G G G G G G G G G . G G G G . G G . G . . . G G G G . . . . G . . . G G G G . . .    
415	C C C C C C C C C C C C C C C C C . C C C C . C C . C . . . C C C C . . . . C . . . C C C C . . .    
416	. A A A A A A A A A A A A A A A A . A A A A . A A . A . . . A A A A . . . . A . . . A A A A . . .    
417	. T T T T T T T T T T T T T T T T . T T T T . T T . T . . . T T T T . . . . T . . . T T T T . . .    
418	. T T T T T T T T T T T T T T T T . T T T T . T T . T . . . T T T T . . . . T . . . T T T T . . .    
419	. G G G G G G G G G G G G G G G G . G G G G . G G . G . . G G G G G . . . . G . . . G G G G . . .    
420	. G G G G G G G G G G G G G G G G . G G G G . G G . G . . G G G G G . . . . G . . . G G G G . . .    
421	. G G G G G G G G G G G G G G G G . G G G G . G G . G . . G G G G G . . . . G . . . G G G G . . .    
422	. A A A A A A A A A A A A A A A A . A A A A . A A . A . . A A A A A . . . . A . . . A A A A . . .    
423	. G G G G G G G G G G G G G G G G . G G G G . G G . G . . G G G G G . . . . G . . . G G G G . . .    
424	. C C C C C C C C C C C C C C C C . C C C C . C C . C . . C C C C C . . . . C . . . C C C C . . .    
425	. A A A A A A A A A A A A A A A A . A A A A . A A . A . . A A A A A . . . . A . . . A A A A . . .    
426	. A A A A A A A A A A A A A A A A . A A A A . A A . A . . A A A A A . . . . A . . . A A A A . . .    
427	. A A A A A A A A A A A A A A A A . A A A A . A A . A . . A A A A A . . . . A . . . A A A A . . .    
428	. C C C C C C C C C C C C C C C C . C C C C . C C . C . . C C C C C . . . . C . . . C C C C . . .    
429	. C C C C C C C C C C C C C C C C . C C C C . C C . C . . C C C C C . . . . C . . . C C C C . . .    
430	. T T T T T T T T T T T T T T T T . T T T T . T T . T . . T T T T T . . . . T . . . T T T T . . .    
431	. C C C C C C C C C C C C C C C C . C C C C . C C . C . C C C C C C . . . . C . . . C C C C . . .    
432	. T T T T T T T T T T T T T T T T . T T T T . T T . T . T T T T T T . . . . T . . . T T T T . . .    
433	. T T T T T T T T T T T T T T T T . T T T T . T T . T . T T T T T T . . . . T . . . T T T T . . .    
434	. C C C C C C C C C C C C C C C C . C C C C . C C . C . C C C C C C . . . . C . . . C C C C . . .    
435	. A A A A A A A A A A A A A A A A . A A A A . A A . A . A A A A A A . . . . A . . . A A A A . . .    
436	. G G G G G G G G G G G G G G G G . G G G G . G G . G . G G G G G G . . . . G . . . G G G G . . .    
437	. A A A A A A A A A A A A A A A A . A A A A . A A . A . A A A A A A . . . . A . . . A A A A . . .    
438	. T T T T T T T T T T T T T T T T . T T T T . T T . T . T T T T T T . . . . T . . . T T T T . . .    
439	. A A A A A A A A A A A A A A A A . A A A A . A A . A . A A A A A A . . . . A . . . A A A A . . .    
440	. T T T T T T T T T T T T T T T T . T T T T . T T . T . T T T T T T . . . . T . . . T T T T . . .    
441	. G G G G G G G G G G G G G G G G . G G G G . G G . G . G G G G G G . . . . G . . . G G G G . . .    
442	. G G G G G G G G G G G G G G G G . G G G G . G G . G . G G G G G G . . . . G . . . G G G G . . .    
443	. G G G G G G G G G G G G G G G G . G G G G . G G . G . G G G G G G . . . . G . . . G G G G . . .    
444	. A A A A A A A A A A A A A A A A A A A A A . A A . A . A A A A A A . . . . A . . . A A A A . . .    
445	. T T T T T T T T T T T T T T T T T T T T T . T T . T . T T T T T T . . . . T T . . T T T T . . .    
446	. A A A A A A A A A A A A A A A A A A A A A . A A . A . A A A A A A . . . . A A . . A A A A . . .    
447	. A A A A A A A A A A A A A A A A A A A A A . A A . A . A A A A A A . . . . A A . . A A A A . . .    
448	. A A A A A A A A A A A A A A A A A A A A A . A A . A . A A A A A A . . . . A A . . A A A A . . .    
449	. A A A A A A A A A A A A A A A A A A A A A . A A . A . A A A A A A . . . . A A . . A A A A . . .    
450	. A A A A A A A A A A A A A A A A A A A A A . A A . A . A A A A A A . . . . A A . . A A A A . . .    
451	. G G G G G G G G G G G G G G G G G G G G G . G G . G . G G G G G G . . . . G G . . G G G G . . .    
452	. G G G G G G G G G G G G G G G G G G G G G . G G . G . G G G G G G . . . . G G . . G G G G . . .    
453	. T T T T T T T T T T T T T T T T T T T T T . T T . T . T T T T T T . . . . T T . T T T T T . . .    
454	. C C C C C C C C C C C C C C C C C C C C C . C C . C . C C C C C C . . . . C C . C C C C C . . .    
455	. A A A A A A A A A A A A A A A A A A A A A . A A . A . A A A A A A . . . . A A . A A A A A . . .    
456	. G G G G G G G G G G G G G G G G G G G G G . G G . G . G G G G G G . . . . G G . G G G G G . . .    
457	. G G G G G G G G G G G G G G G G G G G G G . G G . G . G G G G G G . . . . G G . G G G G G . . .    
458	. A A A A A A A A A A A A A A A A A A A A A . A A . A . A A A A A A . . . . A A . A A A A A . . .    
459	. A A A A A A A A A A A A A A A A A A A A A . A A . A . A A A A A A . . . . A A . A A A A A . . .    
460	. C C C C C C C C C C C C C C C C C C C C C . C C . C . C C C C C C . . . . C C . C C C C C . . .    
461	. G G G G G G G G G G G G G G G G G G G G G . G G . G . G G G G G G . . . . G G . G G G G G . . .    
462	. G G G G G G G G G G G G G G G G G G G G G . G G . G . G G G G G G . . . . G G . G G G G G . . .    
463	. C C C C C C C C C C C C C C C C C C C C C . C C . C . C C C C C C . . . . C C C C C C C C . . .    
464	. C C C C C C C C C C C C C C C C C C C C C . C C . C . C C C C C C . . . . C C C C C C C C . . .    
465	. A A A A A A A A A A A A A A A A A A A A A . A A . A . A A A A A A . . . . A A A A A A A A . . .    
466	. T T T T T T T T T T T T T T T T T T T T T . T T . T . T T T T T T . . . . T T T T T T T T . . .    
467	. A A A A A A A A A A A A A A A A A A A A A . A A . A . A A A A A A . . . . A A A A A A A A . . .    
468	. T T T T T T T T T T T T T T T T T T T T T . T T . T . T T T T T T . . . . T T T T T T T T . . .    
469	. A A A A A A A A A A A A A A A A A A A A A . A A . A . A A A A A A . . . . A A A A A A A A . . .    
470	. A A A A A A A A A A A A A A A A A A A A A . A A . A . A A A A A A . . . . A A A A A A A A . . .    
471	. A A A A A A A A A A A A A A A A A A A A A . A A . A . A A A A A A . . . . A A A A A A A A . . .    
472	. G G G G G G G G G G G G G G G G G G G G G . G G . G . G G G G G G . . . . G G G G G G G G . . .    
473	. C C C C C C C C C C C C C C C C C C C C C . C C . C . C C C C C C . . . . C C C C C C C C . . .    
474	. G G G G G G G G G G G G G G G G G G G G G . G G . G . G G G G G G . . . . G G G G G G G G . . .    
475	. G G G G G G G G G G G G G G G G G G G G G . G G . G . G G G G G G . . . . G G G G G G G G . . .    
476	. A A A A A A A A A A A A A A A A A A A A A . A A . A . A A A A A A . . . . A A A A A A A A . . .    
477	. T T T T T T T T T T T T T T T T T T T T T . T T . T . T T T T T T . . . . T T T T T T T T . . .    
478	. T T T T T T T T T T T T T T T T T T T T T . T T . T . T T T T T T . . . . T T T T T T T T . . .    
479	. A A A A A A A A A A A A A A A A A A A A A . A A . A . A A A A A A . . . . A A A A A A A A . . .    
480	. C C C C C C C C C C C C C C C C C C C C C . C C . C . C C C C C C . . . . C C C C C C C C . . .    
481	. A A A A A A A A A A A A A A A A A A A A A . A A . A . A A A A A A . . . . A A A A A A A A . . .    
482	. G G G G G G G G G G G G G G G G G G G G G . G G . G . G G G G G G . . . . G G G G G G G G . . .    
483	. A A A A A A A A A A A A A A A A A A A A A . A A . A . A A A A A A . . . . A A A A A A A A . . .    
484	. C C C C C C C C C C C C C C C C C C C C C . C C . C . C C C C C C . . . C C C C C C C C C . . .    
485	. A A A A A A A A A A A A A A A A A A A A A . A A . A . A A A A A A . . . A A A A A A A A A . . .    
486	. A A A A A A A A A A A A A A A A A A A A A . A A . A . A A A A A A . . . A A A A A A A A A . . .    
487	. C C C C C C C C C C C C C C C C C C C C C . C C . C . C C C C C C . . . C C C C C C C C C . . .    
488	. G G G G G G G G G G G G G G G G G G G G G . G G . G . G G G G G G . . . G G G G G G G G G . . .    
489	. A A A A A A A A A A A A A A A A A A A A A . A A . A . A A A A A A . . . A A A A A A A A A . . .    
490	. T C T T C T T T T T C T T C T T T T T T T . T C . T . T T T T T C . . . T C T T T C C C C . . .  11	 transition  
491	. A A A A A A A A A A A A A A A A A A A A A . A A . A . A A A A A A . . . A A A A A A A A A . . .    
492	. T T T T T T T T T T T T T T T T T T T T T . T T . T . T T T T T T . . . T T T T T T T T T . . .    
493	. T T T T T T T T T T T T T T T T T T T T T . T T . T . T T T T T T . . . T T T T T T T T T . . .    
494	. C C C C C C C C C C C C C C C C C C C C C . C C . C . C C C C C C . . . C C C C C C C C C . . .    
495	. A A A A A A A A A A A A A A A A A A A A A . A A . A . A A A A A A . . . A A A A A A A A A . . .    
496	. G G G G G G G G G G G G G G G G G G G G G . G G . G . G G G G G G . . . G G G G G G G G G . . .    
497	. T T T T T T T T T T T T T T T T T T T T T . T T . T . T T T T T T . . . T T T T T T T T T . . .    
498	. C C C C C C C C C C C C C C C C C C C C C . C C . C . C C C C C C . . . C C C C C C C C C . . .    
499	. G G G G G G G G G G G G G G G G G G G G G . G G . G . G G G G G G . . . G G G G G G G G G . . .    
500	. C C C C C C C C C C C C C C C C C C C C C . C C . C . C C C C C C . . . C C C C C C C C C . . .    
501	. T T T T T T T T T T T T T T T T T T T T T T T T . T . T T T T T T . . . T T T T T T T T T . . .    
502	. G G G G G G G G G G G G G G G G G G G G G G G G . G . G G G G G G . . . G G G G G G G G G . . .    
503	. G G G G G G G G G G G G G G G G G G G G G G G G . G . G G G G G G . . . G G G G G G G G G . . .    
504	. T T T T T T T T T T T T T T T T T T T T T T T T . T . T T T T T T . . . T T T T T T T T T . . .    
505	. G G G G G G G G G G G G G G G G G G G G G G G G . G . G G G G G G . . . G G G G G G G G G . . .    
506	. C C C C C C C C C C C C C C C C C C C C C C C C . C . C C C C C C . . . C C C C C C C C C . . .    
507	. T T T T T T T T T T T T T T T T T T T T T T T T . T . T T T T T T . . . T T T T T T T T T . . .    
508	. T T T T T T T T T T T T T T T T T T T T T T T T . T . T T T T T T . . . T T T T T T T T T . . .    
509	. G G G G G G G G G G G G G G G G G G G G G G G G . G . G G G G G G . . . G G G G G G G G G . . .    
510	. A A A A A A A A A A A A A A A A A A A A A A A A . A . A A A A A A . . . A A A A A A A A A . . .    
511	. A A A A A A A A A A A A A A A A A A A A A A A A . A . A A A A A A . . . A A A A A A A A A . . .    
512	. G G G G G G G G G G G G G G G G G G G G G G G G . G . G G G G G G . . . G G G G G G G G G . . .    
513	. T T T T T T T T T T T T T T T T T T T T T T T T . T . T T T T T T . . . T T T T T T T T T . . .    
514	. G G G G G G G G G G G G G G G G G G G G G G G G . G . G G G G G G . . . G G G G G G G G G . . .    
515	. G G G G G G G G G G G G G G G G G G G G G G G G . G . G G G G G G . . . G G G G G G G G G . . .    
516	. A A A A A A A A A A A A A A A A A A A A A A A A . A . A A A A A A . . . A A A A A A A A A . . .    
517	. G G G G G G G G G G G G G G G G G G G G G G G G . G . G G G G G G . . . G G G G G G G G G . . .    
518	. G G G G G G G G G G G G G G G G G G G G G G G G . G . G G G G G G . . . G G G G G G G G G . . .    
519	. G G G G G G G G G G G G G G G G G G G G G G G G . G . G G G G G G . . . G G G G G G G G G . . .    
520	. T T T T T T T T T T T T T T T T T T T T T T T T . T . T T T T T T . . . T T T T T T T T T . . .    
521	. A A A A A A A A A A A A A A A A A A A A A A A A . A . A A A A A A . . . A A A A A A A A A . . .    
522	. C C C C C C C C C C C C C C C C C C C C C C C C . C . C C C C C C . . . C C C C C C C C C . . .    
523	. A A A A A A A A A A A A A A A A A A A A A A A A . A . A A A A A A . . . A A A A A A A A A . . .    
524	. A A A A A A A A A A A A A A A A A A A A A A A A . A . A A A A A A . . . A A A A A A A A A . . .    
525	. A A A A A A A A A A A A A A A A A A A A A A A A . A . A A A A A A . . . A A A A A A A A A . . .    
526	. T T T T T T T T T T T T T T T T T T T T T T T T . T . T T T T T T . . . T T T T T T T T T . . .    
527	. G G G G G G G G G G G G G G G G G G G G G G G G . G . G G G G G G . . . G G G G G G G G G . . .    
528	. T T T T T T T T T T T T T T T T T T T T T T T T . T . T T T T T T . . . T T T T T T T T T . . .    
529	. C C C C C C C C C C C C C C C C C C C C C C C C . C . C C C C C C . . . C C C C C C C C C . . .    
530	. A A A A A A A A A A A A A A A A A A A A A A A A . A . A A A A A A . . . A A A A A A A A A . . .    
531	. G G G G G G G G G G G G G G G G G G G G G G G G . G . G G G G G G . . . G G G G G G G G G . . .    
532	. C C C C C C C C C C C C C C C C C C C C C C C C . C . C C C C C C . . . C C C C C C C C C . . .    
533	. A A A A A A A A A A A A A A A A A A A A A A A A . A . A A A A A A . . . A A A A A A A A A . . .    
534	. C C C C C C C C C C C C C C C C C C C C C C C C . C . C C C C C C . . . C C C C C C C C C . . .    
535	. A A A A A A A A A A A A A A A A A A A A A A A A . A . A A A A A A . . . A A A A A A A A A . . .    
536	. A A A A A A A A A A A A A A A A A A A A A A A A . A . A A A A A A . . . A A A A A A A A A . . .    
537	. C C C C C C C C C C C C C C C C C C C C C C C C . C . C C C C C C . . . C C C C C C C C C . . .    
538	. C C C C C C C C C C C C C C C C C C C C C C C C . C . C C C C C C . . . C C C C C C C C C . . .    
539	. T T T T T T T T T T T T T T T T T T T T T T T T . T . T T T T T T . . . T T T T T T T T T . . .    
540	. A A A A A A A A A A A A A A A A A A A A A A A A . A . A A A A A A . . . A A A A A A A A A . . .    
541	. C C C C C C C C C C C C C C C C C C C C C C C C . C . C C C C C C . . . C C C C C C C C C . . .    
542	. A A A A A A A A A A A A A A A A A A A A A A A A . A . A A A A A A . . . A A A A A A A A A . . .    
543	. T T T T T T T T T T T T T T T T T T T T T T T T . T . T T T T T T . . . T T T T T T T T T . . .    
544	. C C C C C C C C C C C C C C C C C C C C C C C C . C . C C C C C C . . . C C C C C C C C C . . .    
545	. A A A A A A A A A A A A A A A A A A A A A A A A . A . A A A A A A . . . A A A A A A A A A . . .    
546	. C C C C C C C C C C C C C C C C C C C C C C C C . C . C C C C C C . . . C C C C C C C C C . . .    
547	. T T T T T T T T T T T T T T T T T T T T T T T T . T . T T T T T T . . . T T T T T T T T T . . .    
548	. T T T T T T T T T T T T T T T T T T T T T T T T . T . T T T T T T . . . T T T T T T T T T . . .    
549	. G G G G G G G G G G G G G G G G G G G G G G G G . G . G G G G G G . . . G G G G G G G G G . . .    
550	. T T T T T T T T T T T T T T T T T T T T T T T T . T . T T T T T T . . . T T T T T T T T T . T .    
551	. C C C C C C C C C C C C C C C C C C C C C C C C . C . C C C C C C . . C C C C C C C C C C . C .    
552	. C C C C C C C C C C C C C C C C C C C C C C C C . C . C C C C C C . . C C C C C C C C C C . C .    
553	. G G G G G G G G G G G G G G G G G G G G G G G G . G . G G G G G G . . G G G G G G G G G G . G .    
554	. G G G G G G G G G G G G G G G G G G G G G G G G . G . G G G G G G . . G G G G G G G G G G . G .    
555	. C C C C C C C C C C C C C C C C C C C C C C C C . C . C C C C C C . . C C C C C C C C C C . C .    
556	. A A A A A A A A A A A A A A A A A A A A A A A A . A . A A A A A A . . A A A A A A A A A A . A .    
557	. G G G G G G G G G G G G G G G G G G G G G G G G . G . G G G G G G . . G G G G G G G G G G . G .    
558	. A A A A A A A A A A A A A A A A A A A A A A A A . A . A A A A A A . . A A A A A A A A A A . A .    
559	. C C C C C C C C C C C C C C C C C C C C C C C C . C . C C C C C C . . C C C C C C C C C C . C .    
560	. C C C C C C C C C C C C C C C C C C C C C C C C . C . C C C C C C . . C C C C C C C C C C . C .    
561	. C C C C C C C C C C C C C C C C C C C C C C C C . C . C C C C C C . . C C C C C C C C C C . C .    
562	. G G G G G G G G G G G G G G G G G G G G G G G G . G . G G G G G G . . G G G G G G G G G G . G .    
563	. A A A A A A A A A A A A A A A A A A A A A A A A . A . A A A A A A . . A A A A A A A A A A . A .    
564	. A A A A A A A A A A A A A A A A A A A A A A A A . A . A A A A A A . . A A A A A A A A A A . A .    
565	. G G G G G G G G G G G G G G G G G G G G G G G G . G . G G G G G G . . G G G G G G G G G G . G .    
566	. A A A A A A A A A A A A A A A A A A A A A A A A . A . A A A A A A . . A A A A A A A A A A . A .    
567	. A A A A A A A A A A A A A A A A A A A A A A A A . A . A A A A A A . . A A A A A A A A A A . A .    
568	. G G G G G G G G G G G G G G G G G G G G G G G G . G . G G G G G G . . G G G G G G G G G G . G .    
569	. A A A A A A A A A A A A A A A A A A A A A A A A . A . A A A A A A . . A A A A A A A A A A . A .    
570	. C C C C C C C C C C C C C C C C C C C C C C C C . C . C C C C C C . . C C C C C C C C C C . C .    
571	. A A A A A A A A A A A A A A A A A A A A A A A A . A . A A A A A A . . A A A A A A A A A A . A .    
572	. C C C C C C C C C C C C C C C C C C C C C C C C . C . C C C C C C . . C C C C C C C C C C . C .    
573	. T T T T T T T T T T T T T T T T T T T T T T T T . T . T T T T T T . . T T T T T T T T T T . T .    
574	. T T T T T T T T T T T T T T T T T T T T T T T T . T . T T T T T T . . T T T T T T T T T T . T .    
575	. G G G G G G G G G G G G G G G G G G G G G G G G . G . G G G G G G . . G G G G G G G G G G . G .    
576	. G G G G G G G G G G G G G G G G G G G G G G G G . G . G G G G G G . . G G G G G G G G G G . G .    
577	. C C C C C C C C C C C C C C C C C C C C C C C C . C . C C C C C C . . C C C C C C C C C C . C .    
578	. A A A A A A A A A A A A A A A A A A A A A A A A . A . A A A A A A . . A A A A A A A A A A . A .    
579	. T T T T T T T T T T T T T T T T T T T T T T T T . T . T T T T T T . . T T T T T T T T T T . T .    
580	. C C C C C C C C C C C C C C C C C C C C C C C C . C . C C C C C C . . C C C C C C C C C C . C .    
581	. A A A A A A A A A A A A A A A A A A A A A A A A . A . A A A A A A . . A A A A A A A A A A . A .    
582	. A A A A A A A A A A A A A A A A A A A A A A A A . A . A A A A A A . . A A A A A A A A A A . A .    
583	. A A A A A A A A A A A A A A A A A A A A A A A A . A . A A A A A A . . A A A A A A A A A A . A .    
584	. C C C C C C C C C C C C C C C C C C C C C C C C . C . C C C C C C . . C C C C C C C C C C . C .    
585	. T T T T T T T T T T T T T T T T T T T T T T T T . T . T T T T T T . . T T T T T T T T T T . T .    
586	. C C C C C C C C C C C C C C C C C C C C C C C C . C . C C C C C C . . C C C C C C C C C C . C .    
587	. C C C C C C C C C C C C C C C C C C C C C C C C . C . C C C C C C . . C C C C C C C C C C . C .    
588	. C C C C C C C C C C C C C C C C C C C C C C C C . C . C C C C C C . . C C C C C C C C C C . C .    
589	. G G G G G G G G G G G G G G G G G G G G G G G G . G . G G G G G G . . G G G G G G G G G G . G .    
590	. T T T T T T T T T T T T T T T T T T T T T T T T . T . T T T T T T . . T T T T T T T T T T . T .    
591	. T T T T T T T T T T T T T T T T T T T T T T T T . T . T T T T T T . . T T T T T T T T T T . T .    
592	. T T T T T T T T T T T T T T T T T T T T T T T T . T . T T T T T T . . T T T T T T T T T T . T .    
593	. T T T T T T T T T T T T T T T T T T T T T T T T . T . T T T T T T . . T T T T T T T T T T . T .    
594	. T T T T T T T T T T T T T T T T T T T T T T T T . T . T T T T T T . . T T T T T T T T T T . T .    
595	. G G G G G G G G G G G G G G G G G G G G G G G G . G . G G G G G G . . G G G G G G G G G G . G .    
596	. G G G G G G G G G G G G G G G G G G G G G G G G . G . G G G G G G . . G G G G G G G G G G . G .    
597	. T T T T T T T T T T T T T T T T T T T T T T T T . T . T T T T T T . . T T T T T T T T T T . T .    
598	. T T T T T T T T T T T T T T T T T T T T T T T T . T . T T T T T T . T T T T T T T T T T T . T .    
599	. A A A A A A A A A A A A A A A A A A A A A A A A . A . A A A A A A . A A A A A A A A A A A . A .    
600	. T T T T T T T T T T T T T T T T T T T T T T T T . T . T T T T T T . T T T T T T T T T T T . T .    
601	. G G G G G G G G G G G G G G G G G G G G G G G G . G . G G G G G G . G G G G G G G G G G G . G .    
602	. A A A A A A A A A A A A A A A A A A A A A A A A . A . A A A A A A . A A A A A A A A A A A . A .    
603	. T T T T T T T T T T T T T T T T T T T T T T T T . T . T T T T T T . T T T T T T T T T T T . T .    
604	. C C C C C C C C C C C C C C C C C C C C C C C C . C . C C C C C C . C C C C C C C C C C C . C .    
605	. A A A A A A A A A A A A A A A A A A A A A A A A . A . A A A A A A . A A A A A A A A A A A . A .    
606	. T T T T T T T T T T T T T T T T T T T T T T T T . T . T T T T T T . T T T T T T T T T T T . T .    
607	. A A A A A A A A A A A A A A A A A A A A A A A A . A . A A A A A A . A A A A A A A A A A A . A .    
608	. A A A A A A A A A A A A A A A A A A A A A A A A . A . A A A A A A . A A A A A A A A A A A . A .    
609	. A A A A A A A A A A A A A A A A A A A A A A A A . A . A A A A A A . A A A A A A A A A A A . A .    
610	. G G G G G G G G G G G G G G G G G G G G G G G G . G . G G G G G G . G G G G G G G G G G G . G .    
611	. A A A A A A A A A A A A A A A A A A A A A A A A . A . A A A A A A . A A A A A A A A A A A . A .    
612	. A A A A A A A A A A A A A A A A A A A A A A A A . A . A A A A A A . A A A A A A A A A A A . A .    
613	. T T T T T T T T T T T T T T T T T T T T T T T T . T . T T T T T T . T T T T T T T T T T T . T .    
614	. T T T T T T T T T T T T T T T T T T T T T T T T . T . T T T T T T . T T T T T T T T T T T . T .    
615	. T T T T T T T T T T T T T T T T T T T T T T T T . T . T T T T T T . T T T T T T T T T T T . T .    
616	. G G G G G G G G G G G G G G G G G G G G G G G G . G . G G G G G G . G G G G G G G G G G G . G .    
617	. A A A A A A A A A A A A A A A A A A A A A A A A A A . A A A A A A . A A A A A A A A A A A . A .    
618	. A A A A A A A A A A A A A A A A A A A A A A A A A A . A A A A A A . A A A A A A A A A A A . A .    
619	. G G G G G G G G G G G G G G G G G G G G G G G G G G . G G G G G G . G G G G G G G G G G G . G .    
620	. A A A A A A A A A A A A A A A A A A A A A A A A A A . A A A A A A . A A A A A A A A A A A . A .    
621	. A A A A A A A A A A A A A A A A A A A A A A A A A A . A A A A A A . A A A A A A A A A A A . A .    
622	. G G G G G G G G G G G G G G G G G G G G G G G G G G . G G G G G G . G G G G G G G G G G G . G .    
623	. T T T T T T T T T T T T T T T T T T T T T T T T T T . T T T T T T . T T T T T T T T T T T . T .    
624	. A A A A A A A A A A A A A A A A A A A A A A A A A A . A A A A A A . A A A A A A A A A A A . A .    
625	. T T T T T T T T T T T T T T T T T T T T T T T T T T . T T T T T T . T T T T T T T T T T T . T .    
626	. T T T T T T T T T T T T T T T T T T T T T T T T T T . T T T T T T . T T T T T T T T T T T . T .    
627	. T T T T T T T T T T T T T T T T T T T T T T T T T T . T T T T T T . T T T T T T T T T T T . T .    
628	. C C C C C C C C C C C C C C C C C C C C C C C C C C . C C C C C C . C C C C C C C C C C C . C .    
629	. A A A A A A A A A A A A A A A A A A A A A A A A A A . A A A A A A . A A A A A A A A A A A . A .    
630	. C C C C C C C C C C C C C C C C C C C C C C C C C C . C C C C C C . C C C C C C C C C C C . C .    
631	. C C C C C C C C C C C C C C C C C C C C C C C C C C . C C C C C C . C C C C C C C C C C C . C .    
632	. T T T T T T T T T T T T T T T T T T T T T T T T T T . T T T T T T . T T T T T T T T T T T . T .    
633	. T T T T T T T T T T T T T T T T T T T T T T T T T T . T T T T T T . T T T T T T T T T T T . T .    
634	. T T T T T T T T T T T T T T T T T T T T T T T T T T . T T T T T T . T T T T T T T T T T T . T .    
635	. G G G G G G G G G G G G G G G G G G G G G G G G G G . G G G G G G . G G G G G G G G G G G . G .    
636	. A A A A A A A A A A A A A A A A A A A A A A A A A A . A A A A A A . A A A A A A A A A A A . A .    
637	. A A A A A A A A A A A A A A A A A A A A A A A A A A . A A A A A A . A A A A A A A A A A A . A .    
638	. G G G G G G G G G G G G G G G G G G G G G G G G G G . G G G G G G . G G G G G G G G G G G . G .    
639	. T T T T T T T T T T T T T T T T T T T T T T T T T T . T T T T T T . T T T T T T T T T T T . T .    
640	. C C C C C C C C C C C C C C C C C C C C C C C C C C . C C C C C C . C C C C C C C C C C C . C .    
641	. C C C C C C C C C C C C C C C C C C C C C C C C C C . C C C C C C . C C C C C C C C C C C . C .    
642	. A A A A A A A A A A A A A A A A A A A A A A A A A A . A A A A A A . A A A A A A A A A A A . A .    
643	. G G G G G G G G G G G G G G G G G G G G G G G G G G . G G G G G G . G G G G G G G G G G G . G .    
644	. G G G G G G G G G G G G G G G G G G G G G G G G G G . G G G G G G . G G G G G G G G G G G . G .    
645	. T T T T T T T T T T T T T T T T T T T T T T T T T T . T T T T T T . T T T T T T T T T T T . T .    
646	. G G G G G G G G G G G G G G G G G G G G G G G G G G . G G G G G G . G G G G G G G G G G G . G .    
647	. T T T T T T T T T T T T T T T T T T T T T T T T T T . T T T T T T . T T T T T T T T T T T . T .    
648	. T T T T T T T T T T T T T T T T T T T T T T T T T T . T T T T T T . T T T T T T T T T T T . T .    
649	. G G G G G G G G G G G G G G G G G G G G G G G G G G . G G G G G G . G G G G G G G G G G G . G .    
650	. G G G G G G G G G G G G G G G G G G G G G G G G G G . G G G G G G . G G G G G G G G G G G . G .    
651	. A A A A A A A A A A A A A A A A A A A A A A A A A A . A A A A A A . A A A A A A A A A A A . A .    
652	. T T T T T T T T T T T T T T T T T T T T T T T T T T . T T T T T T . T T T T T T T T T T T . T .    
653	. G G G G G G G G G G G G G G G G G G G G G G G G G G . G G G G G G . G G G G G G G G G G G . G .    
654	. A A A A A A A A A A A A A A A A A A A A A A A A A A . A A A A A A . A A A A A A A A A A A . A .    
655	. C C C C C C C C C C C C C C C C C C C C C C C C C C . C C C C C C . C C C C C C C C C C C . C .    
656	. A A A A A A A A A A A A A A A A A A A A A A A A A A . A A A A A A . A A A A A A A A A A A . A .    
657	. A A A A A A A A A A A A A A A A A A A A A A A A A A . A A A A A A . A A A A A A A A A A A . A .    
658	. G G G G G G G G G G G G G G G G G G G G G G G G G G . G G G G G G . G G G G G G G G G G G . G .    
659	. A A A A A A A A A A A A A A A A A A A A A A A A A A . A A A A A A . A A A A A A A A A A A . A .    
660	. A A A A A A A A A A A A A A A A A A A A A A A A A A . A A A A A A . A A A A A A A A A A A . A .    
661	. T T T T T T T T T T T T T T T T T T T T T T T T T T . T T T T T T . T T T T T T T T T T T . T .    
662	. G G G G G G G G G G G G G G G G G G G G G G G G G G . G G G G G G . G G G G G G G G G G G . G .    
663	. T T T T T T T T T T T T T T T T T T T T T T T T T T . T T T T T T . T T T T T T T T T T T . T .    
664	. G G G G G G G G G G G G G G G G G G G G G G G G G G . G G G G G G . G G G G G G G G G G G . G .    
665	. A A A A A A A A A A A A A A A A A A A A A A A A A A . A A A A A A . A A A A A A A A A A A . A .    
666	. G G G G G G G G G G G G G G G G G G G G G G G G G G . G G G G G G . G G G G G G G G G G G . G .    
667	. G G G G G G G G G G G G G G G G G G G G G G G G G G . G G G G G G . G G G G G G G G G G G . G .    
668	. A A A A A A A A A A A A A A A A A A A A A A A A A A . A A A A A A . A A A A A A A A A A A . A .    
669	. G G G G G G G G G G G G G G G G G G G G G G G G G G . G G G G G G . G G G G G G G G G G G . G .    
670	. G G G G G G G G G G G G G G G G G G G G G G G G G G . G G G G G G . G G G G G G G G G G G . G .    
671	. C C C C C C C C C C C C C C C C C C C C C C C C C C . C C C C C C . C C C C C C C C C C C . C .    
672	. G G G G G G G G G G G G G G G G G G G G G G G G G G . G G G G G G . G G G G G G G G G G G . G .    
673	. G G G G G G G G G G G G G G G G G G G G G G G G G G . G G G G G G . G G G G G G G G G G G . G .    
674	. C T T T T T T T T T T T T T T T T T T T T T T T T T . T T T T T T . T T T T T T T T T T T . T .    
675	. . T T T T T T T T T T T T T T T T T T T T T T T T T . T T T T T T . T T T T T T T T T T T . T .    
676	. . C C C C C C C C C C C C C C C C C C C C C C C C C . C C C C C C . C C C C C C C C C C C . C .    
677	. . C C C C C C C C C C C C C C C C C C C C C C C C C . C C C C C C . C C C C C C C C C C C . C .    
678	. . G G G G G G G G G G G G G G G G G G G G G G G G G . G G G G G G . G G G G G G G G G G G . G .    
679	. . G G G G G G G G G G G G G G G G G G G G G G G G G . G G G G G G . G G G G G G G G G G G . G .    
680	. . G G G G G G G G G G G G G G G G G G G G G G G G G . G G G G G G . G G G G G G G G G G G . G .    
681	. . C C C C C C C C C C C C C C C C C C C C C C C C C . C C C C C C . C C C C C C C C C C C . C .    
682	. . C C C C C C C C C C C C C C C C C C C C C C C C C . C C C C C C . C C C C C C C C C C C . C .    
683	. . A A A A A A A A A A A A A A A A A A A A A A A A A . A A A A A A . A A A A A A A A A A A . A .    
684	. . G G G G G G G G G G G G G G G G G G G G G G G G G . G G G G G G . G G G G G G G G G G G . G .    
685	. . T T T T T T T T T T T T T T T T T T T T T T T T T . T T T T T T . T T T T T T T T T T T . T .    
686	. . A A A A A A A A A A A A A A A A A A A A A A A A A . A A A A A A . A A A A A A A A A A A . A .    
687	. . A A A A A A A A A A A A A A A A A A A A A A A A A . A A A A A A . A A A A A A A A A A A . A .    
688	. . C C C C C C C C C C C C C C C C C C C C C C C C C . C C C C C C . C C C C C C C C C C C . C .    
689	. . T T T T T T T T T T T T T T T T T T T T T T T T T . T T T T T T . T T T T T T T T T T T . T .    
690	. . A A A A A A A A A A A A A A A A A A A A A A A A A . A A A A A A . A A A A A A A A A A A . A .    
691	. . C C C C C C C C C C C C C C C C C C C C C C C C C . C C C C C C . C C C C C C C C C C C . C .    
692	. . C C C C C C C C C C C C C C C C C C C C C C C C C . C C C C C C . C C C C C C C C C C C . C .    
693	. . A A A A A A A A A A A A A A A A A A A A A A A A A . A A A A A A . A A A A A A A A A A A . A .    
694	. . G G G G G G G G G G G G G G G G G G G G G G G G G . G G G G G G . G G G G G G G G G G G . G .    
695	. . A A A A A A A A A A A A A A A A A A A A A A A A A . A A A A A A . A A A A A A A A A A A . A .    
696	. . G G G G G G G G G G G G G G G G G G G G G G G G G . G G G G G G . G G G G G G G G G G G . G .    
697	. . C C C C C C C C C C C C C C C C C C C C C C C C C . C C C C C C . C C C C C C C C C C C . C .    
698	. . A A A A A A A A A A A A A A A A A A A A A A A A A . A A A A A A . A A A A A A A A A A A . A .    
699	. . C C C C C C C C C C C C C C C C C C C C C C C C C . C C C C C C . C C C C C C C C C C C . C .    
700	. . C C C C C C C C C C C C C C C C C C C C C C C C C . C C C C C C . C C C C C C C C C C C . C .    
701	. . A A A A A A A A A A A A A A A A A A A A A A A A A . A A A A A A . A A A A A A A A A A A . A .    
702	. . C C C C C C C C C C C C C C C C C C C C C C C C C . C C C C C C . C C C C C C C C C C C . C .    
703	. . T T T T T T T T T T T T T T T T T T T T T T T T T . T T T T T T . T T T T T T T T T T T . T .    
704	. . C C C C C C C C C C C C C C C C C C C C C C C C C . C C C C C C . C C C C C C C C C C C . C .    
705	. . G G G G G G G G G G G G G G G G G G G G G G G G G . G G G G G G . G G G G G G G G G G G . G .    
706	. . G G G G G G G G G G G G G G G G G G G G G G G G G . G G G G G G . G G G G G G G G G G G . G .    
707	. . G G G G G G G G G G G G G G G G G G G G G G G G G . G G G G G G . G G G G G G G G G G G . G .    
708	. . T T T T T T T T T T T T T T T T T T T T T T T T T . T T T T T T . T T T T T T T T T T T . T .    
709	. . G G G G G G G G G G G G G G G G G G G G G G G G G . G G G G G G . G G G G G G G G G G G . G .    
710	. . A A A A A A A A A A A A A A A A A A A A A A A A A . A A A A A A . A A A A A A A A A A A . A .    
711	. . A A A A A A A A A A A A A A A A A A A A A A A A A . A A A A A A . A A A A A A A A A A A . A .    
712	. . A A A A A A A A A A A A A A A A A A A A A A A A A . A A A A A A . A A A A A A A A A A A . A .    
713	. . C C C C C C C C C C C C C C C C C C C C C C C C C . C C C C C C . C C C C C C C C C C C . C .    
714	. . C C C C C C C C C C C C C C C C C C C C C C C C C . C C C C C C . C C C C C C C C C C C . C .    
715	. . C C C C C C C C C C C C C C C C C C C C C C C C C . C C C C C C . C C C C C C C C C C C . C .    
716	. . T T T T T T T T T T T T T T T T T T T T T T T T T . T T T T T T . T T T T T T T T T T T . T .    
717	. . T T T T T T T T T T T T T T T T T T T T T T T T T . T T T T T T . T T T T T T T T T T T . T .    
718	. . C C C C C C C C C C C C C C C C C C C C C C C C C . C C C C C C . C C C C C C C C C C C . C .    
719	. . A A A A A A A A A A A A A A A A A A A A A A A A A . A A A A A A . A A A A A A A A A A A . A .    
720	. . T T T T T T T T T T T T T T T T T T T T T T T T T . T T T T T T . T T T T T T T T T T T . T .    
721	. . C C C C C C C C C C C C C C C C C C C C C C C C C . C C C C C C . C C C C C C C C C C C . C .    
722	. . T T T T T T T T T T T T T T T T T T T T T T T T T . T T T T T T . T T T T T T T T T T T . T .    
723	. . G G G G G G G G G G G G G G G G G G G G G G G G G . G G G G G G . G G G G G G G G G G G . G .    
724	. . T T T T T T T T T T T T T T T T T T T T T T T T T . T T T T T T . T T T T T T T T T T T . T .    
725	. . A A A A A A A A A A A A A A A A A A A A A A A A A . A A A A A A . A A A A A A A A A A A . A .    
726	. . C C C C C C C C C C C C C C C C C C C C C C C C C . C C C C C C . C C C C C C C C C C C . C C    
727	. . C C C C C C C C C C C C C C C C C C C C C C C C C . C C C C C C . C C C C C C C C C C C . C C    
728	. . A A A A A A A A A A A A A A A A A A A A A A A A A . A A A A A A . A A A A A A A A A A A . A A    
729	. . T T T T T T T T T T T T T T T T T T T T T T T T T . T T T T T T . T T T T T T T T T T T . T T    
730	. . G G G G G G G G G G G G G G G G G G G G G G G G G . G G G G G G . G G G G G G G G G G G . G G    
731	. . C C C C C C C C C C C C C C C C C C C C C C C C C . C C C C C C . C C C C C C C C C C C . C C    
732	. . C C C C C C C C C C C C C C C C C C C C C C C C C . C C C C C C . C C C C C C C C C C C . C C    
733	. . C C C C C C C C C C C C C C C C C C C C C C C C C . C C C C C C . C C C C C C C C C C C . C C    
734	. . A A A A A A A A A A A A A A A A A A A A A A A A A . A A A A A A . A A A A A A A A A A A . A A    
735	. . T T T T T T T T T T T T T T T T T T T T T T T T T . T T T T T T . T T T T T T T T T T T . T T    
736	. . G G G G G G G G G G G G G G G G G G G G G G G G G . G G G G G G . G G G G G G G G G G G . G G    
737	. . A A A A A A A A A A A A A A A A A A A A A A A A A . A A A A A A . A A A A A A A A A A A . A A    
738	. . G G G G G G G G G G G G G G G G G G G G G G G G G . G G G G G G . G G G G G G G G G G G . G G    
739	. . G G G G G G G G G G G G G G G G G G G G G G G G G . G G G G G G . G G G G G G G G G G G . G G    
740	. . C C C C C C C C C C C C C C C C C C C C C C C C C . C C C C C C . C C C C C C C C C C C . C C    
741	. . T T T T T T T T T T T T T T T T T T T T T T T T T . T T T T T T . T T T T T T T T T T T . T T    
742	. . G G G G G G G G G G G G G G G G G G G G G G G G G . G G G G G G . G G G G G G G G G G G . G G    
743	. . G G G G G G G G G G G G G G G G G G G G G G G G G . G G G G G G . G G G G G G G G G G G . G G    
744	. . A A A A A A A A A A A A A A A A A A A A A A A A A . A A A A A A . A A A A A A A A A A A . A A    
745	. . C C C C C C C C C C C C C C C C C C C C C C C C C . C C C C C C . C C C C C C C C C C C . C C    
746	. . G G G G G G G G G G G G G G G G G G G G G G G G G . G G G G G G . G G G G G G G G G G G . G G    
747	. . A A A A A A A A A A A A A A A A A A A A A A A A A . A A A A A A . A A A A A A A A A A A . A A    
748	. . C C C C C C C C C C C C C C C C C C C C C C C C C . C C C C C C . C C C C C C C C C C C . C C    
749	. . G G G G G G G G G G G G G G G G G G G G G G G G G . G G G G G G . G G G G G G G G G G G . G G    
750	. . G G G G G G G G G G G G G G G G G G G G G G G G G . G G G G G G . G G G G G G G G G G G . G G    
751	. . C C C C C C C C C C C C C C C C C C C C C C C C C . C C C C C C . C C C C C C C C C C C . C C    
752	. . T T T T T T T T T T T T T T T T T T T T T T T T T . T T T T T T . T T T T T T T T T T T . T T    
753	. . G G G G G G G G G G G G G G G G G G G G G G G G G . G G G G G G . G G G G G G G G G G G . G G    
754	. . G G G G G G G G G G G G G G G G G G G G G G G G G . G G G G G G . G G G G G G G G G G G . G G    
755	. . A A A A A A A A A A A A A A A A A A A A A A A A A . A A A A A A . A A A A A A A A A A A . A A    
756	. . A A A A A A A A A A A A A A A A A A A A A A A A A . A A A A A A . A A A A A A A A A A A . A A    
757	. . C C C C C C C C C C C C C C C C C C C C C C C C C . C C C C C C . C C C C C C C C C C C . C C    
758	. . C C C C C C C C C C C C C C C C C C C C C C C C C . C C C C C C . C C C C C C C C C C C . C C    
759	. . A A A A A A A A A A A A A A A A A A A A A A A A A . A A A A A A . A A A A A A A A A A A . A A    
760	. . G G G G G G G G G G G G G G G G G G G G G G G G G . G G G G G G . G G G G G G G G G G G . G G    
761	. . . A A A A A A A A A A A A A A A A A A A A A A A A . A A A A A A . A A A A A A A A A A A . A A    
762	. . . T T T T T T T T T T T T T T T T T T T T T T T T . T T T T T T . T T T T T T T T T T T . T T    
763	. . . C C C C C C C C C C C C C C C C C C C C C C C C . C C C C C C . C C C C C C C C C C C . C C    
764	. . . C C C C C C C C C C C C C C C C C C C C C C C C . C C C C C C . C C C C C C C C C C C . C C    
765	. . . A A A A A A A A A A A A A A A A A A A A A A A A . A A A A A A . A A A A A A A A A A A . A A    
766	. . . G G G G G G G G G G G G G G G G G G G G G G G G . G G G G G G . G G G G G G G G G G G . G G    
767	. . . T T T T T T T T T T T T T T T T T T T T T T T T . T T T T T T . T T T T T T T T T T T . T T    
768	. . . T T T T T T T T T T T T T T T T T T T T T T T T . T T T T T T . T T T T T T T T T T T . T T    
769	. . . T T T T T T T T T T T T T T T T T T T T T T T T . T T T T T T . T T T T T T T T T T T . T T    
770	. . . A A A A A A A A A A A A A A A A A A A A A A A A . A A A A A A . A A A A A A A A A A A . A A    
771	. . . A A A A A A A A A A A A A A A A A A A A A A A A . A A A A A A . A A A A A A A A A A A . A A    
772	. . . C C C C C C C C C C C C C C C C C C C C C C C C . C C C C C C . C C C C C C C C C C C . C C    
773	. . . T C C C C C C C C C C C C C C C C C C C C C C C . C C C C C C . C C C C C C C C C C C . C C    
774	. . . . T T T T T T T T T T T T T T T T T T T T T T T . T T T T T T . T T T T T T T T T T T . T T    
775	. . . . G G G G G G G G G G G G G G G G G G G G G G G . G G G G G G . G G G G G G G G G G G . G G    
776	. . . . T T T T T T T T T T T T T T T T T T T T T T T . T T T T T T . T T T T T T T T T T T . T T    
777	. . . . C C C C C C C C C C C C C C C C C C C C C C C . C C C C C C . C C C C C C C C C C C . C C    
778	. . . . T T T T T T T T T T T T T T T T T T T T T T T . T T T T T T . T T T T T T T T T T T . T T    
779	. . . . G G G G G G G G G G G G G G G G G G G G G G G . G G G G G G . G G G G G G G G G G G . G G    
780	. . . . A A A A A A A A A A A A A A A A A A A A A A A . A A A A A A . A A A A A A A A A A A . A G    
781	. . . . C C C C C C C C C C C C C C C C C C C C C C C . C C C C C C . C C C C C C C C C C C . C C    
782	. . . . T T T T T T T T T T T T T T T T T T T T T T T . T T T T T T . T T T T T T T T T T T . T T    
783	. . . . T T T T T T T T T T T T T T T T T T T T T T T . T T T T T T . T T T T T T T T T T T . T T    
784	. . . . C C C C C C C C C C C C C C C C C C C C C C C . C C C C C C . C C C C C C C C C C C . C C    
785	. . . . A A A A A A A A A A A A A A A A A A A A A A A . A A A A A A . A A A A A A A A A A A . A A    
786	. . . . C C C C C C C C C C C C C C C C C C C C C C C . C C C C C C . C C C C C C C C C C C . C C    
787	. . . . A A A A A A A A A A A A A A A A A A A A A A A . A A A A A A . A A A A A A A A A A A . A A    
788	. . . . C C C C C C C C C C C C C C C C C C C C C C C . C C C C C C . C C C C C C C C C C C . C C    
789	. . . . G G G G G G G G G G G G G G G G G G G G G G G . G G G G G G . G G G G G G G G G G G . G G    
790	. . . . G G G G G G G G G G G G G G G G G G G G G G G . G G G G G G . G G G G G G G G G G G . G G    
791	. . . . C C C C C C C C C C C C C C C C C C C C C C C . C C C C C C . C C C C C C C C C C C . C C    
792	. . . . G G G G G G G G G G G G G G G G G G G G G G G . G G G G G G . G G G G G G G G G G G . G G    
793	. . . . G G G G G G G G G G G G G G G G G G G G G G G . G G G G G G . G G G G G G G G G G G . G G    
794	. . . . G G G G G G G G G G G G G G G G G G G G G G G . G G G G G G . G G G G G G G G G G G . G G    
795	. . . . C C C C C C C C C C C C C C C C C C C C C C C . C C C C C C . C C C C C C C C C C C . C C    
796	. . . . C C C C C C C C C C C C C C C C C C C C C C C . C C C C C C . C C C C C C C C C C C . C C    
797	. . . . T T T T T T T T T T T T T T T T T T T T T T T . T T T T T T . T T T T T T T T T T T . T T    
798	. . . . . A A A A A A A A A A A A A A A A A A A A A A . A A A A A A . A A A A A A A A A A A . A A    
799	. . . . . T T T T T T T T T T T T T T T T T T T T T T . T T T T T T . T T T T T T T T T T T . T T    
800	. . . . . G G G G G G G G G G G G G G G G G G G G G G . G G G G G G . G G G G G G G G G G G . G G    
801	. . . . . G G G G G G G G G G G G G G G G G G G G G G . G G G G G G . G G G G G G G G G G G . G G    
802	. . . . . C C C C C C C C C C C C C C C C C C C C C C . C C C C C C . C C C C C C C C C C C . C C    
803	. . . . . A A A A A A A A A A A A A A A A A A A A A A . A A A A A A . A A A A A A A A A A A . A A    
804	. . . . . C C C C C C C C C C C C C C C C C C C C C C . C C C C C C . C C C C C C C C C C C . C C    
805	. . . . . C C C C C C C C C C C C C C C C C C C C C C . C C C C C C . C C C C C C C C C C C . C C    
806	. . . . . A A A A A A A A A A A A A A A A A A A A A A . A A A A A A . A A A A A A A A A A A . A A    
807	. . . . . A A A A A A A A A A A A A A A A A A A A A A . A A A A A A . A A A A A A A A A A A . A A    
808	. . . . . C C C C C C C C C C C C C C C C C C C C C C . C C C C C C . C C C C C C C C C C C . C C    
809	. . . . . T T T T T T T T T T T T T T T T T T T T T T . T T T T T T . T T T T T T T T T T T . T T    
810	. . . . . A A A A A A A A A A A A A A A A A A A A A A . A A A A A A . A A A A A A A A A A A . A A    
811	. . . . . C C C C C C C C C C C C C C C C C C C C C C . C C C C C C . C C C C C C C C C C C . C C    
812	. . . . . A A A A A A A A A A A A A A A A A A A A A A . A A A A A A . A A A A A A A A A A A . A A    
813	. . . . . T T T T T T T T T T T T T T T T T T T T T T . T T T T T T . T T T T T T T T T T T . T T    
814	. . . . . C C C C C C C C C C C C C C C C C C C C C C . C C C C C C . C C C C C C C C C C C . C C    
815	. . . . . G G G G G G G G G G G G G G G G G G G G G G . G G G G G G . G G G G G G G G G G G . G G    
816	. . . . . A A A A A A A A A A A A A A A A A A A A A A . A A A A A A . A A A A A A A A A A A . A A    
817	. . . . . G G G G G G G G G G G G G G G G G G G G G G . G G G G G G . G G G G G G G G G G G . G G    
818	. . . . . A A A A A A A A A A A A A A A A A A A A A A . A A A A A A . A A A A A A A A A A A . A A    
819	. . . . . C C C C C C C C C C C C C C C C C C C C C C . C C C C C C . C C C C C C C C C C C . C C    
820	. . . . . A A A A A A A A A A A A A A A A A A A A A A . A A A A A A . A A A A A A A A A A A . A A    
821	. . . . . C C C C C C C C C C C C C C C C C C C C C C . C C C C C C . C C C C C C C C C C C . C C    
822	. . . . . T T T T T T T T T T T T T T T T T T T T T T . T T T T T T . T T T T T T T T T T T . T T    
823	. . . . . C C C C C C C C C C C C C C C C C C C C C C . C C C C C C . C C C C C C C C C C C . C C    
824	. . . . . C C C C C C C C C C C C C C C C C C C C C C . C C C C C C . C C C C C C C C C C C . C C    
825	. . . . . G G G G G G G G G G G G G G G G G G G G G G . G G G G G G . G G G G G G G G G G G . G G    
826	. . . . . C C C C C C C C C C C C C C C C C C C C C C . C C C C C C . C C C C C C C C C C C . C C    
827	. . . . . G G G G G G G G G G G G G G G G G G G G G G . G G G G G G . G G G G G G G G G G G . G G    
828	. . . . . T T T T T T T T T T T T T T T T T T T T T T . T T T T T T . T T T T T T T T T T T . T T    
829	. . . . . A A A A A A A A A A A A A A A A A A A A A A . A A A A A A . A A A A A A A A A A A . A A    
830	. . . . . C C C C C C C C C C C C C C C C C C C C C C . C C C C C C . C C C C C C C C C C C . C C    
831	. . . . . A A A A A A A A A A A A A A A A A A A A A A . A A A A A A . A A A A A A A A A A A . A A    
832	. . . . . G G G G G G G G G G G G G G G G G G G G G G . G G G G G G . G G G G G G G G G G G . G G    
833	. . . . . A A A A A A A A A A A A A A A A A A A A A A . A A A A A A . A A A A A A A A A A A . A A    
834	. . . . . T T T T T T T T T T T T T T T T T T T T T T . T T T T T T . T T T T T T T T T T T . T T    
835	. . . . . C C C C C C C C C C C C C C C C C C C C C C . C C C C C C . C C C C C C C C C C C . C C    
836	. . . . . C C C C C C C C C C C C C C C C C C C C C C . C C C C C C . C C C C C C C C C C C . C C    
837	. . . . . A A A A A A A A A A A A A A A A A A A A A A . A A A A A A . A A A A A A A A A A A . A A    
838	. . . . . T T T T T T T T T T T T T T T T T T T T T T . T T T T T T . T T T T T T T T T T T . T T    
839	. . . . . G G G G G G G G G G G G G G G G G G G G G G . G G G G G G . G G G G G G G G G A G . G G    
840	. . . . . C C C C C C C C C C C C C C C C C C C C C C . C C C C C C . C C C C C C C C C C C . C C    
841	. . . . . A A A A A A A A A A A A A A A A A A A A A A . A A A A A A . A A A A A A A A A A A . A A    
842	. . . . . A A A A A A A A A A A A A A A A A A A A A A . A A A A A A . A A A A A A A A A A A . A A    
843	. . . . . A A A A A A A A A A A A A A A A A A A A A A . A A A A A A . A A A A A A A A A A A . A A    
844	. . . . . C C C C C C C C C C C C C C C C C C C C C C . C C C C C C . C C C C C C C C C C C . C C    
845	. . . . . T T T T T T T T T T T T T T T T T T T T T T . T T T T T T . T T T T T T T T T T T . T T    
846	. . . . . G G G G G G G G G G G G G G G G G G G G G G . G G G G G G . G G G G G G G G G G G . G G    
847	. . . . . C C C C C C C C C C C C C C C C C C C C C C . C C C C C C . C C C C C C C C C C C . C C    
848	. . . . . C C C C C C C C C C C C C C C C C C C C C C . C C C C C C . C C C C C C C C C C C . C C    
849	. . . . . G G G G G G G G G G G G G G G G G G G G G G . G G G G G G . G G G G G G G G G G G . G G    
850	. . . . . C C C C C C C C C C C C C C C C C C C C C C . C C C C C C . C C C C C C C C C C C . C C    
851	. . . . . A A A A A A A A A A A A A A A A A A A A A A . A A A A A A . A A A A A A A A A A A . A A    
852	. . . . . T T T T T T T T T T T T T T T T T T T T T T . T T T T T T . T T T T T T T T T T T . T T    
853	. . . . . A A A A A A A A A A A A A A A A A A A A A A . A A A A A A . A A A A A A A A A A A . A A    
854	. . . . . C C C C C C C C C C C C C C C C C C C C C C . C C C C C C . C C C C C C C C C C C . C C    
855	. . . . . G G G G G G G G G G G G G G G G G G G G G G . G G G G G G . G G G G G G G G G G G . G G    
856	. . . . . G G G G G G G G G G G G G G G G G G G G G G . G G G G G G . G G G G G G G G G G G . G G    
857	. . . . . A A A A A A A A A A A A A A A A A A A A A A . A A A A A A . A A A A A A A A A A A . A A    
858	. . . . . G G G G G G G G G G G G G G G G G G G G G G . G G G G G G . G G G G G G G G G G G . G G    
859	. . . . . A A A A A A A A A A A A A A A A A A A A A A . A A A A A A . A A A A A A A A A A A . A A    
860	. . . . . G G G G G G G G G G G G G G G G G G G G G G . G G G G G G . G G G G G G G G G G G . G G    
861	. . . . . T T T T T T T T T T T T T T T T T T T T T T . T T T T T T . T T T T T T T T T T T . T T    
862	. . . . . G G G G G G G G G G G G G G G G G G G G G G . G G G G G G . G G G G G G G G G G G . G G    
863	. . . . . T T T T T T T T T T T T T T T T T T T T T T . T T T T T T . T T T T T T T T T T T . T T    
864	. . . . . A A A A A A A A A A A A A A A A A A A A A A . A A A A A A . A A A A A A A A A A A . A A    
865	. . . . . C C C C C C C C C C C C C C C C C C C C C C . C C C C C C . C C C C C C C C C C C . C C    
866	. . . . . T T T T T T T T T T T T T T T T T T T T T T . T T T T T T . T T T T T T T T T T T . T T    
867	. . . . . T T T T T T T T T T T T T T T T T T T T T T . T T T T T T . T T T T T T T T T T T . T T    
868	. . . . . C C C C C C C C C C C C C C C C C C C C C C . C C C C C C . C C C C C C C C C C C . C C    
869	. . . . . T T T T T T T T T T T T T T T T T T T T T T . T T T T T T . T T T T T T T T T T T . T T    
870	. . . . . C C C C C C C C C C C C C C C C C C C C C C . C C C C C C . C C C C C C C C C C C . C C    
871	. . . . . T T T T T T T T T T T T T T T T T T T T T T . T T T T T T . T T T T T T T T T T T T T T    
872	. . . . . G G G G G G G G G G G G G G G G G G G G G G G G G G G G G . G G G G G G G G G G G G G G    
873	. . . . . A A A A A A A A A A A A A A A A A A A A A A A A A A A A A . A A A A A A A A A A A A A A    
874	. . . . . C C C C C C C C C C C C C C C C C C C C C C C C C C C C C . C C C C C C C C C C C C C C    
875	. . . . . A A A A A A A A A A A A A A A A A A A A A A A A A A A A A . A A A A A A A A A A A A A A    
876	. . . . . G G G G G G G G G G G G G G G G G G G G G G G G G G G G G . G G G G G G G G G G G G G G    
877	. . . . . A A A A A A A A A A A A A A A A A A A A A A A A A A A A A . G A A A A A A A A A A A A A    
878	. . . . . C C C C C C C C C C C C C C C C C C C C C C C C C C C C C . C C C C C C C C C C C C C C    
879	. . . . . T T T T T T T T T T T T T T T T T T T T T T T T T T T T T . T T T T T T T T T T T T T T    
880	. . . . . G G G G G G G G G G G G G G G G G G G G G G G G G G G G G . G G G G G G G G G G G G G G    
881	. . . . . T T T T T T T T T T T T T T T T T T T T T T T T T T T T T . T T T T T T T T T T T T T T    
882	. . . . . A A A A A A A A A A A A A A A A A A A A A A A A A A A A A . A A A A A A A A A A A A A A    
883	. . . . . C C C C C C C C C C C C C C C C C C C C C C C C C C C C C . C C C C C C C C C C C C C C    
884	. . . . . T T T T T T T T T T T T T T T T T T T T T T T T T T T T T T T T T T T T T T T T T T T T    
885	. . . . . . C C C C C C C C C C C C C C C C C C C C C C C C C C C C C C C C C C C C C C C C C C C    
886	. . . . . . A A A A A A A A A A A A A A A A A A A A A A A A A A A A A A A A A A A A A A A A A A A    
887	. . . . . . G G G G G G G G G G G G G G G G G G G G G G G G G G G G G G G G G G G G G G G G G G G    
888	. . . . . . A A A A A A A A A A A A A A A A A A A A A A A A A A A A A A A A A A A A A A A A A A A    
889	. . . . . . A A A A A A A A A A A A A A A A A A A A A A A A A A A A A A A A A A A A A A A A A A A    
890	. . . . . . G G G G G G G G G G G G G G G G G G G G G G G G G G G G G G G G G G G G G G G G G G G    
891	. . . . . . A A A A A A A A A A A A A A A A A A A A A A A A A A A A A A A A A A A A A A A A A A A    
892	. . . . . . T T T T T T T T T T T T T T T T T T T T T T T T T T T T T T T T T T T T T T T T T T T    
893	. . . . . . G G G G G G G G G G G G G G G G G G G G G G G G G G G G G G G G G G G G G G G G G G G    
894	. . . . . . A A A A A A A A A A A A A A A A A A A A A A A A A A A A A A A A A A A A A A A A A A A    
895	. . . . . . G G G G G G G G G G G G G G G G G G G G G G G G G G G G G G G G G G G G G G G G G G G    
896	. . . . . . C C C C C C C C C C C C C C C C C C C C C C C C C C C C C C C C C C C C C C C C C C C    
897	. . . . . . T T T T T T T T T T T T T T T T T T T T T T T T T T T T T T T T T T T T T T T T T T T    
898	. . . . . . C C C C C C C C C C C C C C C C C C C C C C C C C C C C C C C C C C C C C C C C C C C    
899	. . . . . . C C C C C C C C C C C C C C C C C C C C C C C C C C C C C C C C C C C C C C C C C C C    
900	. . . . . . C C C C C C C C C C C C C C C C C C C C C C C C C C C C C C C C C C C C C C C C C C C    
901	. . . . . . T T T T T T T T T T T T T T T T T T T T T T T T T T T T T T T T T T T T T T T T T T T    
902	. . . . . . G G G G G G G G G G G G G G G G G G G G G G G G G G G G G G G G G G G G G G G G G G G    
903	. . . . . . C C C C C C C C C C C C C C C C C C C C C C C C C C C C C C C C C C C C C C C C C C C    
904	. . . . . . G G G G G G G G G G G G G G G G G G G G G G G G G G G G G G G G G G G G G G G G G G G    
905	. . . . . . G G G G G G G G G G G G G G G G G G G G G G G G G G G G G G G G G G G G G G G G G G G    
906	. . . . . . A A A A A A A A A A A A A A A A A A A A A A A A A A A A A A A A A A A A A A A A A A A    
907	. . . . . . G G G G G G G G G G G G G G G G G G G G G G G G G G G G G G G G G G G G G G G G G G G    
908	. . . . . . T T T T T T T T T T T T T T T T T T T T T T T T T T T T T T T T T T T T T T T T T T T    
909	. . . . . . T T T T T T T T T T T T T T T T T T T T T T T T T T T T T T T T T T T T T T T T T T T    
910	. . . . . . C C C C C C C C C C C C C C C C C C C C C C C C C C C C C C C C C C C C C C C C C C C    
911	. . . . . . A A A A A A A A A A A A A A A A A A A A A A A A A A A A A A A A A A A A A A A A A A A    
912	. . . . . . A A A A A A A A A A A A A A A A A A A A A A A A A A A A A A A A A A A A A A A A A A G    
913	. . . . . . A A A A A A A A A A A A A A A A A A A A A A A A A A A A A A A A A A A A A A A A A A A    
914	. . . . . . C C C C C C C C C C C C C C C C C C C C C C C C C C C C C C C C C C C C C C C C C C C    
915	. . . . . . T T T T T T T T T T T T T T T T T T T T T T T T T T T T T T T T T T T T T T T T T T T    
916	. . . . . . T T T T T T T T T T T T T T T T T T T T T T T T T T T T T T T T T T T T T T T T T T T    
917	. . . . . . T T T T T T T T T T T T T T T T T T T T T T T T T T T T T T T T T T T T T T T T T T T    
918	. . . . . . A A A A A A A A A A A A A A A A A A A A A A A A A A A A A A A A A A A A A A A A A A A    
919	. . . . . . T T T T T T T T T T T T T T T T T T T T T T T T T T T T T T T T T T T T T T T T T T T    
920	. . . . . . C C C C C C C C C C C C C C C C C C C C C C C C C C C C C C C C C C C C C C C C C C C    
921	. . . . . . T T T T T T T T T T T T T T T T T T T T T T T T T T T T T T T T T T T T T T T T T T T    
922	. . . . . . . A A A A A A A A A A A A A A A A A A A A A A A A A A A A A A A A A A A A A A A A A A    
923	. . . . . . . C C C C C C C C C C C C C C C C C C C C C C C C C C C C C C C C C C C C C C C C C C    
924	. . . . . . . C T C C C C C C C C C C C C C C C C C C C C C C C C C C C C C C C C C C C C C C C C    
925	. . . . . . . C . C C C C C C C C C C C C C C C C C C C C C C C C C C C C C C C C C C C C C C C C    
926	. . . . . . . G . G G G G G G G G G G G G G G G G G G G G G G G G G G G G G G G G G G G G G G G G    
927	. . . . . . . T . T T T T T T T T T T T T T T T T T T T T T T T T T T C T T T T T T T T T T T T T    
928	. . . . . . . T . T T T T T T T T T T T T T T T T T T T T T T T T T T T T T T T T T T T T T T T T    
929	. . . . . . . C . C C C C C C C C C C C C C C C C C C C C C C C C C C C C C C C C C C C C C C C C    
930	. . . . . . . A . A A A A A A A A A A A A A A A A A A A A A A A A A A A A A A A A A A A A A A A A    
931	. . . . . . . G . G G G G G G G G G G G G G G G G G G G G G G G G G G G G G G G G G G G G G G G G    
932	. . . . . . . A . A A A A A A A A A G A A A A A A A A A A A A A A A A A A A A A A A A A A A A A A    
933	. . . . . . . A . A A A A A A A A A A A A A A A A A A A A A A A A A A A A A A A A A A A A A A A A    
934	. . . . . . . C . C C C C C C C C C C C C C C C C C C C C C C C C C C C C C C C C C C C C C C C C    
935	. . . . . . . A . A A A A A A A A A A A A A A A A A A A A A A A A A A A A A A A A A A A A A A A A    
936	. . . . . . . A . A A A A A A A A A A A A A A A A A A A A A A A A A A A A A A A A A A A A A A A A    
937	. . . . . . . A . A A A A A A A A A A A A A A A A A A A A A A A A A A A A A A A A A A A A A A A A    
938	. . . . . . . G . G G G G G G G G G G G G G G G G G G G G G G G G G G G G G G G G G G G G G G G G    
939	. . . . . . . C . C C C C C C C C C C T T C C C C C C C C C C C C C C C C C C C C C C C C C C C C    
940	. . . . . . . A . A A A A A A A A A A A A A A A A A A A A A A A A A A A A A A A A A A A A A A A A    
941	. . . . . . . A . A A A A A A A A A A A A A A A A A A A A A A A A A A A A A A A A A A A A A A A A    
942	. . . . . . . A . A A A A A A A A A A A A A A A A A A A A A A A A A A A A A A A A A A A A A A A A    
943	. . . . . . . G . G G G G G G G G G G G G G G G G G G G G G G G G G G G G G G G G G G G G G G G G    
944	. . . . . . . T . C C C - - - C C C - - - C - - C C C C C C C C C C C C C - - - - - - - C C C C C  15	 indel  
945	. . . . . . . A . A A A - - - A A A - - - A - - A A A A A A A A A A A A A - - - - - - - A A A A A  15	 indel  
946	. . . . . . . A . G G G - - - G G G - - - G - - G G G G G G G G G G G G G - - - - - - - G G G G G  15	 indel  
947	. . . . . . . A . T T T T T T T T T T T T T T T T T T T T T T T T T T T T T T T T T T T T T T T T    
948	. . . . . . . . . A A A A A A A A A A A A A A A A A A A A A A A A A A A A A A A A A A A A A A A A    
949	. . . . . . . . . A A A A A A A A A A A A A A A A A A A A A A A A A A A A A A A A A A A A A A A A    
950	. . . . . . . . . G G G G G G G G G G G G G G G G G G G G G G G G G G G G G G G G G G G G G G G G    
951	. . . . . . . . . G G G G G G G G G G G G G G G G G G G G G G G G G G G G G G G G G G G G G G G G    
952	. . . . . . . . . C C C C C C C C C C C C C C C C C C C C C C C C C C C C C C C C C C C C C C C C    
953	. . . . . . . . . A A A A A A A A A A A A A A A A A A A A A A A A A A A A A A A A A A A A A A A A    
954	. . . . . . . . . G G G G G G G G G G G G G G G G G G G G G G G G G G G G G G G G G G G G G G G G    
955	. . . . . . . . . C C C C C C C C C C C C C C C C C C C C C C C C C C C C C C C C C C C C C C C C    
956	. . . . . . . . . T T T T T T T T T T T T T T T T T T T T T T T T T T T T T T T T T T T T T T T T    
957	. . . . . . . . . G G G G G G G G G G G G G G G G G G G G G G G G G G G G G G G G G G G G G G G G    
958	. . . . . . . . . A A A A A A A A A A A A A A A A A A A A A A A A A A A A A A A A A A A A A A A A    
959	. . . . . . . . . A A A A A A A A A A A A A A A A A A A A A A A A A A A A A A A A A A A A A A A A    
960	. . . . . . . . . T T T T T T T T T T T T T T T T T T T T T T T T T T T T T T T T T T T T T T T T    
961	. . . . . . . . . C C C C C C C C C C C C C C C C C C C C C C C C C C C C C C C C C C C C C C C C    
962	. . . . . . . . . G . G G G G G G G G G G G G G G G G G G G G G G G G G G G G G G G G G G G G G G    
963	. . . . . . . . . A . A A A A A A A A A A A A A A A A A A A A A A A A A A A A A A A A A A A A A A    
964	. . . . . . . . . T . T T T T T T T T T T T T T T T T T T T T T T T T T T T T T T T T T T T T T T    
965	. . . . . . . . . T . T T T T T T T T T T T T T T T T T T T T T T T T T T T T T T T T T T T T T T    
966	. . . . . . . . . C . C T C C C C C C C C C C C C C C C C C C C C C C C C C C C C C C C C C C C C    
967	. . . . . . . . . T . T . T T T T T T T T T T T T T T T T T T T T T T T T T T T T T T T T T T T T    
968	. . . . . . . . . C . C . C C C C C C C C C C C C C C C C C C C C C C C C C C C C C C C C C C C C    
969	. . . . . . . . . C . C . C C C C C C C C C C C C C C C C C C C C C C C C C C C C C C C C C C C C    
970	. . . . . . . . . T . T . T T T T T T T T T T T T T T T T T T T T T T T T T T T T T T T T T T T T    
971	. . . . . . . . . C . C . C C C C C C C C C C C C C C C C C C C C C C C C C C C C C C C C C C C C    
972	. . . . . . . . . C . C . C C C C C C C C C C C C C C C C C C C C C C C C C C C C C C C C C C C C    
973	. . . . . . . . . C . C . C C C C C C C C C C C C C C C C C C C C C C C C C C C C C C C C C C C C    
974	. . . . . . . . . C . C . C C C C C C C C C C C C C C C C C C C C C C C C C C C C C C C C C C C C    
975	. . . . . . . . . A . A . A A A A A A A A A A A A A A A A A A A A A A A A A A A A A A A A A A A A    
976	. . . . . . . . . T . T . T T T T T T T T T T T T T T T T T T T T T T T T T T T T T T T T T T T T    
977	. . . . . . . . . T . T . T T T T T T T T T T T T T T T T T T T T T T T T T T T T T T T T T T T T    
978	. . . . . . . . . C . C . C C C C C C C C C C C C C C C C C C C C C C C C C C C C C C C C C C C C    
979	. . . . . . . . . C . C . C C C C C C C C C C C C C C C C C C C C C C C C C C C C C C C C C C C C    
980	. . . . . . . . . A . A . A A A A A A A A A A A A A A A A A A A A A A A A A A A A A A A A A A A A    
981	. . . . . . . . . G . G . G G G G G G G G G G G G G G G G G G G G G G G G G G G G G G G G G G G G    
982	. . . . . . . . . A . A . A A A A A A A A A A A A A A A A A A A A A A A A A A A A A A A A A A A A    
983	. . . . . . . . . T . T . T T T T T T T T T T T T T T T T T T T T T T T T T T T T T T T T T T T T    
984	. . . . . . . . . G . G . G G G G G G G G G G G G G G G G G G G G G G G G G G G G G G G G G G G G    
985	. . . . . . . . . T . T . T T T T T T T T T T T T C T T T T T T T T T T T T T T T T T T T T T T T    
986	. . . . . . . . . G . G . G G G G G G G G G G G G G G G G G G G G G G G G G G G G G G G G G G G G    
987	. . . . . . . . . A . A . A A A A A A A A A A A A A A A A A A A A A A A A A A A A A A A A A A A A    
988	. . . . . . . . . A . A . A A A A A A A A A A A A A A A A A A A A A A A A A A A A A A A A A A A A    
989	. . . . . . . . . T . T . T T T T T T T T T T T T T T T T T T T T T T T T T T T T T T T T T T T T    
990	. . . . . . . . . A . A . A A A A A A A A A A A A A A A A A A A A A A A A A A A A A A A A A A A A    
991	. . . . . . . . . T . T . T T T T T T T T T T T T T T T T T T T T T T T T T T T T T T T T T T T T    
992	. . . . . . . . . T . T . T T T T T T T T T T T T T T T T T T T T T T T T T T T T T T T T T T T T    
993	. . . . . . . . . A . A . A A A A A A A A A A A A A A A A A A A A A A A A A A A A A A A A A A A A    
994	. . . . . . . . . T . T . T T T T T T T T T T T T T T T T T T T T T T T T T T T T T T T T T T T T    
995	. . . . . . . . . A . A . A A A A A A A A A A A A A A A A A A A A A A A A A A A A A A A A A A A A    
996	. . . . . . . . . T . T . T T T T T T T T T T T T T T T T T T T T T T T T T T T T T T T T T T T T    
997	. . . . . . . . . T . T . T T T T T T T T T T T T T T T T T T T T T T T T T T T T T T T T T T T T    
998	. . . . . . . . . T . T . T T T T T T T T T T T T T T T T T T T T T T T T T T T T T T T T T T T T    
999	. . . . . . . . . G . G . G G G G G G G G G G G G G G G G G G G G G G G G G G G G G G G G G G G G    
1000	. . . . . . . . . C . C . C C C C C C C C C C C C C C C C C C C C C C C C C C C C C C C C C C C C    
1001	. . . . . . . . . A . A . A A A A A A A A A A A A A A A A A A A A A A A A A A A A A A A A A A A A    
1002	. . . . . . . . . G . G . G G G G G G G G G G G G G G G G G G G G G G G G G G G G G G G G G G G G    
1003	. . . . . . . . . A . A . A A A A A A A A A A A A A A A A A A A A A A A A A A A A A A A A A A A A    
1004	. . . . . . . . . T . T . T T T T T T T T T T T T T T T T T T T T T T T T T T T T T T T T T T T T    
1005	. . . . . . . . . A . A . A A A A A A A A A A A A A A A A A A A A A A A A A A A A A A A A A A A A    
1006	. . . . . . . . . G . G . G G G G G G G G G G G G G G G G G G G G G G G G G G G G G G G G G G G G    
1007	. . . . . . . . . T . T . T T T T T T T T T T T T T T T T T T T T T T T T T T T T T T T T T T T T    
1008	. . . . . . . . . C . C . C C C C C C C C C C C C C C C C C C C C C C C C C C C C C C C C C C C C    
1009	. . . . . . . . . T . T . T T T T T T T T T T T T T T T T T T T T T T T T T T T T T T T T T T T T    
1010	. . . . . . . . . A . A . A A A A A A A A A A A A A A A A A A A A A A A A A A A A A A A A A A A A    
1011	. . . . . . . . . C . C . C C C C C C C C C C C C C C C C C C C C C C C C C C C C C C C C C C C C    
1012	. . . . . . . . . A . A . A A A A A A A A A A A A A A A A A A A A A A A A A A A A A A A A A A A A    
1013	. . . . . . . . . T . . . T T T T T T T T T T T T T T T T T T T T T T T T T T T T T T T T T T T T    
1014	. . . . . . . . . T . . . T T T T T T T T T T T T T T T T T T T T T T T T T T T T T T T T T T T T    
1015	. . . . . . . . . T . . . T T T T T T T T T T T T T T T T T T T T T T T T T T T T T T T T T T T T    
1016	. . . . . . . . . . . . . G G G G G G G G G G G G G G G G G G G G G G G G G G G G G G G G G G G G    
1017	. . . . . . . . . . . . . T T T T T T T T T T T T T T T T T T T T T T T T T T T T T T T T T T T T    
1018	. . . . . . . . . . . . . T T T T T T T T T T T T T T T T T T T T T T T T T T T T T T T T T T T T    
1019	. . . . . . . . . . . . . G G G G G G G G G G G G G G G G G G G G G G G G G G G G G G G G G G G G    
1020	. . . . . . . . . . . . . T T T T T T T T T T T T T T T T T T T T T T T T T T T T T T T T T T T T    
1021	. . . . . . . . . . . . . A A A A A A A A A A A A A A A A A A A A A A A A A A A A A A A A A A A A    
1022	. . . . . . . . . . . . . A A A A A A A A A A A A A A A A A A A A A A A A A A A A A A A A A A A A    
1023	. . . . . . . . . . . . . T T T T T T T T T T T T T T T T T T T T T T T T T T T T T T T T T T T T    
1024	. . . . . . . . . . . . . G G G G G G G G G G G G G G G G G G G G G G G G G G G G G G G G G G G G    
1025	. . . . . . . . . . . . . T T T T T T T T T T T T T T T T T T T T T T T T T T T T T T T T T T T T    
1026	. . . . . . . . . . . . . T T T T T T T T T T T T T T T T T T T T T T T T T T T T T T T T T T T T    
1027	. . . . . . . . . . . . . T T T T T T T T T T T T T T T T T T T T T T T T T T T T T T T T T T T T    
1028	. . . . . . . . . . . . . T T T T T T T T T T T T T T T T T T T T T T T T T T T T T T T T T T T T    
1029	. . . . . . . . . . . . . T T T T T T T T T T T T T T T T T T T T T T T T T T T T T T T T T T T T    
1030	. . . . . . . . . . . . . G G G G G G G G G G G G G G G G G G G G G G G G G G G G G G G G G G G G    
1031	. . . . . . . . . . . . . . C C C C C C C C C C C C C C C C C C C C C C C C C C C C C C C C C C C    
1032	. . . . . . . . . . . . . . T T T T T T T T . T T T T T T T T T T T T T T T T T T T T T T T T T T    
1033	. . . . . . . . . . . . . . T . T T T T T T . T T T T T T T T T T T T T T T T T T T T T T T T T T    
1034	. . . . . . . . . . . . . . T . T T T T T T . T T T T T T T T T T T T T T T T T T T T T T T T T T    
1035	. . . . . . . . . . . . . . G . G G G G G G . G G G G G G G G G G G G G G G G G G G G G G G G G G    
1036	. . . . . . . . . . . . . . G . G G G G G G . G G G G G G G G G G G G G G G G G G G G G G G G G G    
1037	. . . . . . . . . . . . . . A . A A A A A A . A A A A A A A A A A A A A A A A A A A A A A A A A A    
1038	. . . . . . . . . . . . . . A . A A A A A A . A A A A A A A A A A A A A A A A A A A A A A A A A A    
1039	. . . . . . . . . . . . . . C . C C C C C C . C C C C C C C C C C C C C C C C C C C C C C C C C C    
1040	. . . . . . . . . . . . . . C . C C C C C C . C C C C C C C C C C C C C C C C C C C C C C C C C C    
1041	. . . . . . . . . . . . . . A . A A A A A A . A A A A A A A A A A A A A A A A A A A A A A A A A A    
1042	. . . . . . . . . . . . . . T . T T T T T T . T T T T T T T T T T T T T T T T T T T T T T T T T T    
1043	. . . . . . . . . . . . . . G . G G G G G G . G G G G G G G G G G G G G G G G G G G G G G G G G G    
1044	. . . . . . . . . . . . . . C . C C C C C C . C C C C C C C C C C C C C C C C C C C C C C C C C C    
1045	. . . . . . . . . . . . . . G . G G G G G G . G G G G G G G G G G G G G G G G G G G G G G G G G G    
1046	. . . . . . . . . . . . . . A . . A A A A A . A A A A A A A A A A A A A A A A A A A A A A A A A A    
1047	. . . . . . . . . . . . . . T . . T T T T T . T T T T T T T T T T T T T T T T T T T T T T T T T T    
1048	. . . . . . . . . . . . . . T . . T T T T T . T T T T T T T T T T T T T T T T T T T T T T T T T T    
1049	. . . . . . . . . . . . . . G . . G G G G G . G G G G G G G G G G G G G G G G G G G G G G G G G G    
1050	. . . . . . . . . . . . . . T . . T T T T T . T T T T T T T T T T T T T T T T T T T T T T T T T T    
1051	. . . . . . . . . . . . . . T . . T T T T T . T T T T T T T T T T T T T T T T T T T T T T T T T T    
1052	. . . . . . . . . . . . . . T . . T T T T T . T T T T T T T T T T T T T T T T T T T T T T T T T T    
1053	. . . . . . . . . . . . . . T . . T T T T T . T T T T T T T T T T T T T T T T T T T T T T T T T T    
1054	. . . . . . . . . . . . . . C . . C C C C C . C C C C C C C C C C C C C C C C C C C C C C C C C C    
1055	. . . . . . . . . . . . . . T . . T T T T T . T T T T T T T T T T T T T T T T T T T T T T T T T T    
1056	. . . . . . . . . . . . . . C . . C C C C C . C C C C C C C C C C C C C C C C C C C C C C C C C C    
1057	. . . . . . . . . . . . . . T . . T T T T T . T T T T T T T T T T T T T T T T T T T T T T T T T T    
1058	. . . . . . . . . . . . . . G . . G G G G G . G G G G G G G G G G G G G G G G G G G G G G G G G G    
1059	. . . . . . . . . . . . . . C . . C C C C C . C C C C C C C C C C C C C C C C C C C C C C C C C C    
1060	. . . . . . . . . . . . . . A . . A A A A A . A A A A A A A A A A A A A A A A A A A A A A A A A A    
1061	. . . . . . . . . . . . . . C . . C C C C C . C C C C C C C C C C C C C C C C C C C C C C C C C C    
1062	. . . . . . . . . . . . . . . . . A A A A A . A A A A A A A A A A A A A A A A A A A A A A A A A A    
1063	. . . . . . . . . . . . . . . . . A . . . A . . A A A A A A A A A A A A A A A A A A A A A A A A A    
1064	. . . . . . . . . . . . . . . . . T . . . T . . T T T T T T T T T T T T T T T T T T T T T T T T T    
1065	. . . . . . . . . . . . . . . . . A . . . A . . A A A A A A A A A A A A A A A A A A A A A A A A A    
1066	. . . . . . . . . . . . . . . . . A . . . A . . A A A A A A A A A A A A A A A A A A A A A A A A A    
1067	. . . . . . . . . . . . . . . . . A . . . A . . A A A A A A A A A A A A A A A A A A A A A A A A A    
1068	. . . . . . . . . . . . . . . . . C . . . C . . C C C C C C C C C C C C C C C C C C C C C C C C C    
1069	. . . . . . . . . . . . . . . . . A . . . A . . A A A A A A A A A A A A A A A A A A A A A A A A A    
1070	. . . . . . . . . . . . . . . . . T . . . . . . T T T T T T T T T T T T T T T T T T T T T T T T T    
1071	. . . . . . . . . . . . . . . . . T . . . . . . T T T T T T T T T T T T T T T T T T T T T T T T T    
1072	. . . . . . . . . . . . . . . . . T . . . . . . T T T T T T T T T T T T T T T T T T T T T T T T T    
1073	. . . . . . . . . . . . . . . . . T . . . . . . T T T T T T T T T T T T T T T T T T T T T T T T T    
1074	. . . . . . . . . . . . . . . . . G . . . . . . G G G G G G G G G G G G G G G G G G G G G G G G G    
1075	. . . . . . . . . . . . . . . . . T . . . . . . T T T T T T T T T T T T T T T T T T T T T T T T T    
1076	. . . . . . . . . . . . . . . . . A . . . . . . A A A A A A A A A A A A A A A A A A A A A A A A A    
1077	. . . . . . . . . . . . . . . . . T . . . . . . T T T T T T T T T T T T T T T T T T T T T T T T T    
1078	. . . . . . . . . . . . . . . . . G . . . . . . G G G G G G G G G G G G G G G G G G G G G G G G G    
1079	. . . . . . . . . . . . . . . . . C . . . . . . C C C C C C C C C C C C C C C C C C C C C C C C C    
1080	. . . . . . . . . . . . . . . . . . . . . . . . . . A A A A A A A A A A A A A A A A A A A A A A A    
1081	. . . . . . . . . . . . . . . . . . . . . . . . . . C C C C C C C C C C C C C C C C C C C C C C C    
1082	. . . . . . . . . . . . . . . . . . . . . . . . . . . . . . . . . A . . . . . . . . . . . A A A A    
1083	. . . . . . . . . . . . . . . . . . . . . . . . . . . . . . . . . G . . . . . . . . . . . G G G G    
1084	. . . . . . . . . . . . . . . . . . . . . . . . . . . . . . . . . T . . . . . . . . . . . T T T T    
1085	. . . . . . . . . . . . . . . . . . . . . . . . . . . . . . . . . T . . . . . . . . . . . T C T T    
1086	. . . . . . . . . . . . . . . . . . . . . . . . . . . . . . . . . . . . . . . . . . . . . . . . A    
1087	. . . . . . . . . . . . . . . . . . . . . . . . . . . . . . . . . . . . . . . . . . . . . . . . A    
1088	. . . . . . . . . . . . . . . . . . . . . . . . . . . . . . . . . . . . . . . . . . . . . . . . A    
1089	. . . . . . . . . . . . . . . . . . . . . . . . . . . . . . . . . . . . . . . . . . . . . . . . G    
1090	. . . . . . . . . . . . . . . . . . . . . . . . . . . . . . . . . . . . . . . . . . . . . . . . G
```
